# Electron Transfer Interactome of Cytochrome *c*

**DOI:** 10.1371/journal.pcbi.1002807

**Published:** 2012-12-06

**Authors:** Alexander N. Volkov, Nico A. J. van Nuland

**Affiliations:** 1 Jean Jeener NMR Centre, Structural Biology Brussels, Vrije Universiteit Brussel, Brussels, Belgium; 2 Department of Structural Biology, VIB, Brussels, Belgium; UNC Charlotte, United States of America

## Abstract

Lying at the heart of many vital cellular processes such as photosynthesis and respiration, biological electron transfer (ET) is mediated by transient interactions among proteins that recognize multiple binding partners. Accurate description of the ET complexes – necessary for a comprehensive understanding of the cellular signaling and metabolism – is compounded by their short lifetimes and pronounced binding promiscuity. Here, we used a computational approach relying solely on the steric properties of the individual proteins to predict the ET properties of protein complexes constituting the functional interactome of the eukaryotic cytochrome *c* (Cc). Cc is a small, soluble, highly-conserved electron carrier protein that coordinates the electron flow among different redox partners. In eukaryotes, Cc is a key component of the mitochondrial respiratory chain, where it shuttles electrons between its reductase and oxidase, and an essential electron donor or acceptor in a number of other redox systems. Starting from the structures of individual proteins, we performed extensive conformational sampling of the ET-competent binding geometries, which allowed mapping out functional epitopes in the Cc complexes, estimating the upper limit of the ET rate in a given system, assessing ET properties of different binding stoichiometries, and gauging the effect of domain mobility on the intermolecular ET. The resulting picture of the Cc interactome 1) reveals that most ET-competent binding geometries are located in electrostatically favorable regions, 2) indicates that the ET can take place from more than one protein-protein orientation, and 3) suggests that protein dynamics within redox complexes, and not the electron tunneling event itself, is the rate-limiting step in the intermolecular ET. Further, we show that the functional epitope size correlates with the extent of dynamics in the Cc complexes and thus can be used as a diagnostic tool for protein mobility.

## Author Summary

A number of vital cellular processes such as respiration, photosynthesis, and multifarious metabolic conversions rely on a long-range electron transfer (ET) among protein molecules. Full understanding of the biological ET requires accurate description of the redox protein complexes, which is hampered by their pronounced mobility and short lifetimes. Here we used a simple computational approach to predict the ET properties of the physiological protein complexes of cytochrome *c* (Cc) – a small electron carrier that coordinates the electron flow among different redox partners. By performing extensive conformational sampling of the possible binding geometries, we mapped out functional epitopes in the Cc complexes and assessed their ET properties. Our study suggests that protein dynamics within redox complexes is the rate-limiting step in the intermolecular ET and indicates that the functional epitope size correlates with the extent of dynamics in the Cc complexes. We believe that the latter finding can be used as a diagnostic tool for protein mobility and expect that this work will engender future studies of the intermolecular ET in biological networks.

## Introduction

Many vital cellular processes such as photosynthesis, respiration, and a variety of metabolic reactions rely on long-range electron transfer (ET) between macromolecules. In a biological context, the intermolecular ET involves formation of a redox complex, electron tunneling across a biomolecular interface, and dissociation of the reduced and oxidized products. While the electron tunneling is, arguably, the best described step in this sequence of events [1], [2], our understanding of the first and the last steps is often obscured by the macromolecular dynamics and the uncertainty of the binding geometries in reactive complexes [3]. Experimental study of the biomolecular ET in solution is further complicated by two factors. First, in order to maximize the overall turnover in the ET chain, redox complexes have short lifetimes and, consequently, low binding affinities, making structural characterization of these weak, transient, highly dynamic complexes difficult [4]. Second, unlike in other biomolecular interactions where formation of a functional complex requires precise alignment of the partner molecules, an efficient intermolecular ET can occur from more than one protein-protein orientation. This possible multitude of active binding geometries presents an additional challenge for an accurate description of the biomolecular ET.

The observation that most of the transient ET complexes are formed by oppositely-charged proteins spurred numerous modeling studies, the earliest of which aimed at optimizing intermolecular electrostatic interactions and minimizing the separation between the binding partners' redox cofactors [5], [6]. These were followed by more elaborate simulations [7]–[11], which revealed regions of attractive electrostatic potential containing a range of favorable protein-protein orientations [7]–[9], [12] or indicated possible binding-induced conformational changes [7], [11]. However, crystal structures of ET protein complexes show very few charge-charge or polar contacts across the interfaces, with most of the interactions dominated by van der Waals (vdW) forces [13]. Moreover, in several systems, mutagenesis of the charged residues expected to stabilize transient protein-protein complexes shows little or no effect on the binding affinity and the ET rate [14]–[16]. Thus, contrary to the conclusions of early modeling studies [5], [6], it appears that ET complexes are not optimized for intermolecular electrostatic interactions, which is thought to facilitate rapid dissociation required for a high turnover [13]. The current view is that charged residues – often located at the periphery of the binding sites – play a critical role in the early stages of protein-protein interactions by steering binding partners to form a loose encounter complex, but are less important for the ensuing formation of the specific, ET-active form, dominated by short-range forces [4], [13], [17], [18]. In addition, electrostatic interactions can guide the partner proteins to productive docking geometries, without contributing to the overall binding affinity [19].

The discrepancies between the experimental results and the outcome of the electrostatics-based simulations highlight the need for alternative computational approaches. In this work, we used a modeling protocol based solely on steric properties of protein molecules to describe the ET network of eukaryotic cytochrome *c* (Cc) – a small, soluble, highly conserved hemoprotein. Cc is a key component of the eukaryotic respiratory chain, where it shuttles electrons between Cc reductase (Cbc_1_) and Cc oxidase (CCO) [20]. Its other physiological redox partners include Cc peroxidase (CcP) [21], sulfite oxidase (SOX) [22], flavocytochrome *b*_2_ (Fcb_2_) [23], a flavin-dependent sulfhydryl oxidase Erv1 [24], and possibly cytochrome *b*_5_ (Cb_5_) [20], [25], [26]. In each of these systems, Cc acts as an electron donor or acceptor and serves as a hub coordinating the electron flow in the mitochondrial intermembrane space [26]. Starting from the structures of individual proteins, we performed extensive conformational sampling of possible ET-active binding geometries, which allowed mapping out functional epitopes in the Cc complexes, estimating the upper limit of the ET rate in a given system, assessing ET properties of different binding stoichiometries and, in the case of Cc complexes with SOX and Fcb_2_, gauging the effect of domain mobility on the intermolecular ET.

## Methods

### Molecular Modeling

Protein coordinates were taken from the PDB; cofactor topologies and parameters were obtained from the HIC-Up database [27]. The ScErv1 structure was modeled with Modeller [28] based on the X-ray coordinates of the homologous protein AtErv1 [29]. Missing protein loops were built with Modeller [28]. Hydrogen atoms were added in Xplor-NIH using standard topology and parameter sets [30]. The ensembles of Fcb_2_ and SOX domain-domain orientations were generated in Xplor-NIH by simulated annealing in torsion angle space with standard protein geometry and vdW parameters and knowledge-based dihedral angle potentials [31]. Two domains were treated as rigid bodies, while the intervening linker segment (residues 99–100 or 89–99 for Fcb_2_; and residues 83–94 for SOX) was allowed full flexibility. Detailed description of the systems studied is provided in the Table S1.

### Conformational Search

**Figure 1:**
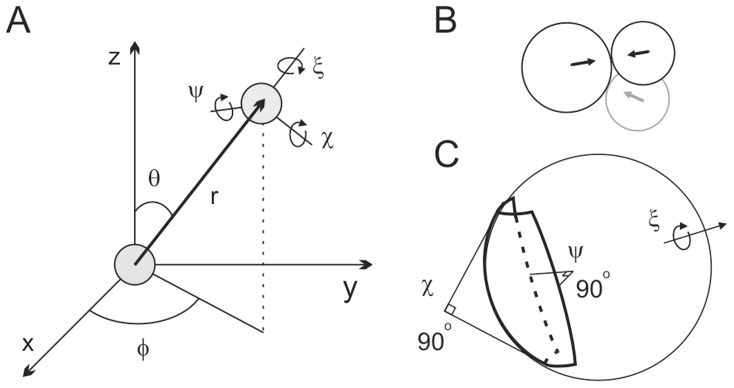
Illustration of the conformational search used in this work. (A) Definition of the search space in the spherical coordinates. Proteins' CMs are shown as grey spheres, one of which is at the origin of the coordinate system. The moving protein (typically Cc) possesses three translational (θ, φ, *r*) and three rotational (χ, ψ, ξ) degrees of freedom. For the definition of the rotational axes see text. (B, C) Conformational search in the reduced rotational space. (B) Cc in the starting structure (black circles) is rotated to obtain the shortest separation between the ET cofactors (arrows). This ensures the frontal orientation of the Cc heme during subsequent θ and φ rotations (grey circle). (C) The reduced rotational space −45°≤χ, ψ≤45° traced by a point on the surface of a sphere (bold shape). Location of the other rotation axis, ξ, is indicated.

Conformational sampling was performed in Xplor-NIH [30] using a modified version of the published protocol [32]. The molecular system was oriented such that the centers of mass (CM) of the two interacting proteins appeared at the origin of the coordinate system and on the positive z axis. In a typical run, the Cc binding partner was kept stationary, while Cc was systematically rotated around x and z axes, corresponding to the θ and φ rotations in the spherical coordinate space (Figure 1A). The desired spatial resolution, δd – defined here as the separation between neighboring CMs of the rotated protein and typically set to 1 Å – determines the δθ and δφ rotation increments and the total number of steps required to sample the entire conformational space (Equations 1–3).
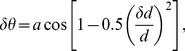
(1)where δd and d are the desired spatial resolution and the distance between CMs of the two proteins in the starting structure. Number of δθ rotational increments is given by n_θ_ = π/δθ, with the value rounded to the last digit before the decimal so that 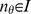. For θ rotation around the x axis, the rotation matrix is given by:
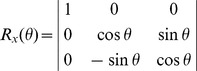
(2)Upon θ rotation, the ordinate of the Cc CM becomes y′ = d·sinθ. By symmetry with the Equation 1, the δφ_i_ rotational increment at each θ_i_ is given by:
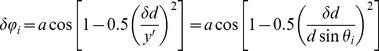
(3)At each θ_i_, the number of δφ_i_ rotational increments is given by n_φ,i_ = 2π/δφ_i_, with the value rounded to the last digit before the decimal so that 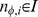. For φ rotation around the z axis, the rotation matrix is given by:
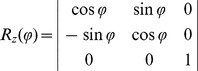
(4)The total number of the CMs defining the coverage of the conformational space is given by
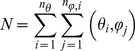
(5)To sample flat, irregular surfaces of the selected CCO and Cbc_1_ regions, the protocol was modified to include the initial translation of Cc along the long axis of the molecular system, followed by rotation around the same axis, both in increments required to produce 1 Å separation between neighboring Cc CMs.

To simulate the rotational freedom, Cc was rotated around orthogonal χ, ψ, ξ axes originating at its CM (Figure 1A). The rotational axes were defined relative to the plane determined by the two proteins' CMs and the Fe atom of Cc with the corresponding Cartesian coordinates (*x*_0_, *y*_0_, *z*_0_), (*x*_1_, *y*_1_, *z*_1_), and (*x*_2_, *y*_2_, *z*_2_) (Equation 6):
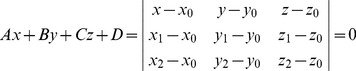
(6)Solving the determinant and noticing that one of the CMs is at the origin of the coordinate system, i.e. (*x*_0_, *y*_0_, *z*_0_) = (0, 0, 0), gives A = *y*_1_ *z*_2_−*y*_2_ *z*_1_, B = *x*_2_ *z*_1_−*x*_1_ *z*_2_, C = *x*_1_ *y*_2_−*y*_1_ *x*_2_, and D = 0. The χ and ξ axes are defined along the (A, B, C) normal to the plane and the (*x*_1_, *y*_1_, *z*_1_) vector joining the two CMs, respectively, while the φ axis is perpendicular to the latter two and is given by the vector (A′, B′, C′) = (C*y*_1_−B*z*_1_, A*z*_1_−C*x*_1_, B*x*_1_−A*y*_1_).

Two rotational sampling protocols were employed in this work. In the first protocol, the rotational coordinates were systematically varied in δχ = δψ = δξ increments in the full rotational space (0°≤χ,ψ<360°, 0°≤ξ<180°). In the second protocol, the conformational search was performed with composite rotation increments (δχ = δψ = 5°, δξ = 15°) in the reduced rotational space (−45°≤χ,ψ≤45°, 0°≤ξ<360°) (Figure 1B,C). In the latter case, the Cc in the starting protein-protein orientation was rotated around its CM in δχ = δψ = δξ = 5° increments (0°≤χ,ψ<360°, 0°≤ξ<180°) in the search for the position with the smallest separation between the ET cofactors (black circles in Figure 1B). In this way, the frontal orientation of the Cc heme was ensured during subsequent θ and φ rotations (grey circle in Figure 1B), enabling sampling of short intermolecular ET distances during the reduced rotational search (Figure 1C).

**Figure 2:**
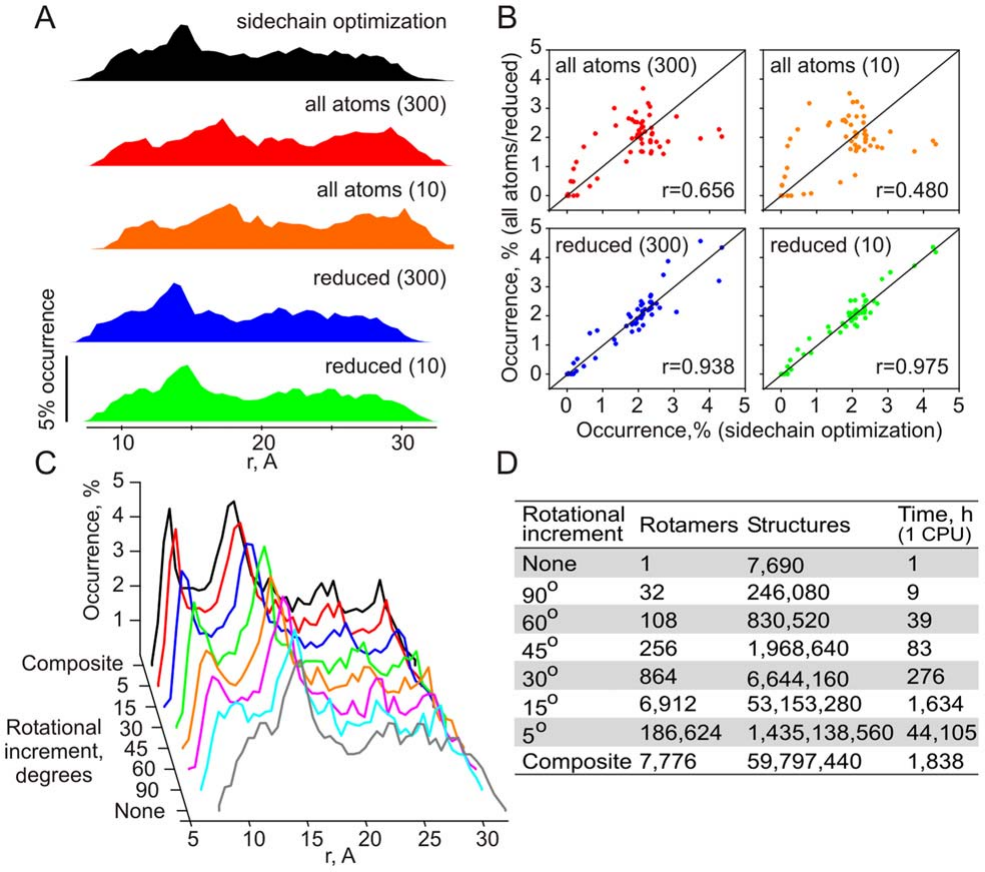
Conformational sampling in the Cc-Cb_5_ complex. (A) Distribution of the heme-heme distances in the simulation runs with the full vdW potential and side-chain optimization (black); full vdW potential without side-chain optimization (vdW cut-off values of 300 kcal/mol and 10 kcal/mol; red and orange, respectively); and reduced vdW term, set to zero for all atoms extending beyond C_β_ (vdW cut-off values 300 kcal/mol and 10 kcal/mol; blue and green, respectively). In these runs, only translational degrees of freedom of Cc were simulated. (B) Correlation plots of heme-heme distance occurrences in the side-chain optimization run (horizontal axis) and the other control runs (vertical axis), color-coded as in (A). Pearson correlation coefficients are indicated in the plots. (C) Distribution of the heme-heme distances in the runs with the reduced vdW term (vdW cut-off 10 kcal/mol, green dataset in A) and a given rotational increment (δχ = δψ = δξ, 0°≤χ, ψ<360°, 0°≤ξ<180°). For the composite run, δχ = δψ = 5°, δξ = 15°; −45°≤χ, ψ≤45°, 0°≤ξ<360°. (D) Number of rotamers for each Cc CM, the total number of structures sampled per run, and the total run time (for 1 CPU).

For each rotamer, the intermolecular vdW energy term (*f_vdW_*) was calculated with a repulsive quartic potential (Equation 7) [33], [34]:
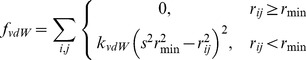
(7)where *r_ij_* is the interatomic distance, *r_min_* adopts the values of the standard vdW radii as represented by the Lennard-Jones potential [33], s is the scaling factor (the value of 0.75, frequently used in Xplor rigid-body docking [33], [34], was employed throughout), and *k_vdW_* is a force-constant (20 kcal·mol^−1^Å^−4^). The sum is carried over all pairwise intermolecular interactions between the atoms that are below a specified non-bonded cut-off, typically set to a value between 5.5 and 8.5 Å. The vdW potential was set to zero for all side-chain atoms extending beyond C_β_, and Cc translated along the vector joining two proteins' CMs until the vdW energy reached the value between zero and a chosen cut-off, thus producing a rigid-body mimic of the protein complex. The separation between the proteins' CMs determines the other translational coordinate, *r*. In control runs, side-chain optimization with full vdW potential was performed after each translational step (Figure 2). For each (χ, ψ, ξ) set, the smallest edge-to-edge distance between the redox cofactors, d_min_, was calculated, and the rotamer with the smallest d_min_ selected. The d_min_ at each sampled (θ, φ) position was used in the subsequent distance distribution analysis and the ET-rate calculations.

In the Cc-CCO system, the proximity of the CCO residue W104 to the protein surface necessitated side-chain optimization of the final docking solutions. Simulations of ternary complexes were performed by sampling binding geometries of the second Cc molecule disregarding the Cc bound in the 1∶1 complex and subsequent evaluation of the vdW term between the two Cc molecules. Only the solutions with the vdW terms below a defined cut-off were retained. A similar approach was used for modeling Cc binding to the multidomain proteins Fcb_2_ and SOX: the interaction of Cc with the Fcb_2_ or SOX heme domain was followed by evaluation of the overall vdW energies and selection of the allowed solutions. The annotated Xplor-NIH scripts for the conformational sampling are provided in the Dataset S1; their detailed description is given in the Text S1.

### ET Rate Calculations

The ET rate constants were calculated from Equation 8 [2]:
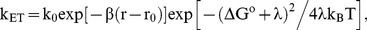
(8)where k_0_ = 10^13^ s^−1^ is the nuclear frequency, β = 1.4 Å^−1^ is the decay coefficient of the electronic coupling, r is the edge-to-edge distance between the redox centers, r_0_ = 3.6 Å is the vdW contact distance, ΔG^o^ is the free energy difference between reactant and product states, λ is the reorganization energy, k_B_ is the Boltzmann constant, and T is the temperature. The values of ΔG^o^ and λ were taken from the literature (see Text S2).

## Results/Discussion

### Conformational Sampling

To define the conformational space available to the interacting molecules and determine the shortest distance between redox cofactors at each sampled point, Cc was used as a probe to explore the surface of a partner protein in search of possible binding geometries. Proteins were treated as rigid bodies with van der Waals (vdW) potential set to zero for all atoms extending beyond C_β_ to allow partial side-chain overlap, mimicking the uncertainty of the side-chain positions due to local binding-induced reorganization. Steric properties of the generated complexes were assessed by calculating the intermolecular vdW energy term.

To determine the optimal parameter set for the conformational search protocol, we performed a number of control runs (Figure 2). In the first control series (where only translational freedom of Cc was simulated to accelerate the calculations), we examined the choice of the vdW cut-offs (Figure 2A,B). The value of 10 kcal/mol, found to yield realistic mimics of protein complexes in our earlier work [32], and a more permissive 300 kcal/mol cut-off were assessed. First, we performed a run with the full vdW potential and side-chain optimization for the entire complex at each translation step. The ET distance distribution in this run (black profile in Figure 2A) was used as a reference for evaluating the protocols without side-chain optimization and the full vdW potential (vdW cut-off values of 300 kcal/mol and 10 kcal/mol; red and orange, respectively in Figure 2A,B) or a reduced vdW term, set to zero for all atoms extending beyond C_β_ (vdW cut-off values 300 kcal/mol and 10 kcal/mol; blue and green, respectively in Figure 2A,B). As can be seen in Figure 2A, the red and orange profiles are shifted towards higher *r* values, indicating that the rigid-body simulations with the full vdW potential produce binding geometries with unrealistically large separations between protein molecules. This is further evidenced by poor correlations between the corresponding distance distributions (top plots in Figure 2B). The runs with the reduced vdW term (blue and green in Figure 2A) exhibit profiles highly similar to that of the reference run and feature greatly improved linear trends in the correlation plots (bottom graphs in Figure 2B). In particular, an excellent agreement between the results of the reference run and the computationally faster simulation protocol with the reduced vdW potential and the cut-off value of 10 kcal/mol (green datasets in Figure 2A,B) indicates that the latter produces reasonable rigid-body models of protein complexes. Therefore, this protocol was used in all subsequent simulations.

Having established the optimal vdW parameters, we proceeded to evaluate the choice of rotational increments to be used in the conformational search protocol. As can be seen in Figure 2C, including Cc rotations around orthogonal axes centered on its CM allows sampling shorter ET distances, with progressively shorter distances accessed at decreasing rotational increments. This is accompanied by a steady increase in the number of rotamers, reaching ca. 187,000 per (θ, φ) position for δχ = δψ = δξ = 5° (Figure 2D). Decreasing the rotational increments further quickly leads to an explosion of structures (e.g. 1.8·10^11^ structures per run with δχ = δψ = δξ = 1°), rendering the computations prohibitively time-consuming. Besides, the number of shorter distances accessed is expected to reach a plateau at smaller increments, making the choice of δχ = δψ = δξ = 5° a good compromise between the density of the rotational space sampling and the computational time invested.

Finally, we noticed that in protein-protein complexes with short ET distances, Cc is oriented with its heme group facing the binding partner (Figure 1B). In addition, control runs with the stationary Cc and Cb_5_ or CcP as the sampling probes showed that the Cc functional epitope maps onto a well-defined patch of the protein surface surrounding the exposed heme edge (Figures S6 and S7). Based on these observations, we devised an accelerated sampling scheme in a reduced rotational space, consisting of defining the frontal Cc orientation in the starting structure, usual sampling of θ and φ dimensions, and the rotational search in a reduced χ, ψ, ξ space. As can be seen in Figure 2C (compare red and black traces), the ET distance distributions obtained with the full sampling scheme (0°≤χ,ψ<360°, 0°≤ξ<180°; δχ = δψ = δξ = 5°) and the reduced rotational search (−45°≤χ, ψ≤45°, 0°≤ξ<360°; δχ = δψ = 5°, δξ = 15°) are virtually identical. At the same time, confining rotations to a smaller space of −30°≤χ, ψ≤30°, 0°≤ξ<360° leads to reduced occurrences of small ET distances, indicating insufficient sampling of the rotational space. Thus, the former protocol – affording a 24-fold gain in the computational time (Figure 2D) – was used in all subsequent simulations.

**Figure 3:**
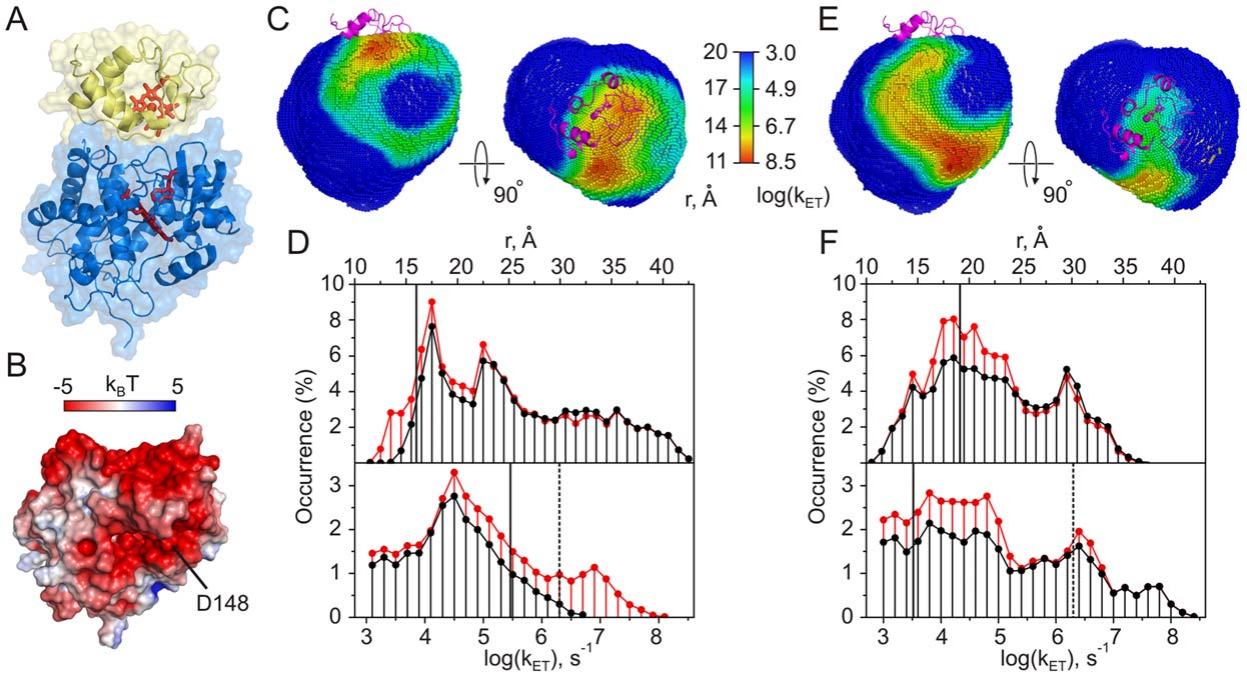
Cc-CcP. (A) Crystal structure of the complex [37]. Cc and CcP are in yellow and blue, respectively; heme groups and CcP residue W191 are shown as red sticks. (B) The molecular surface of CcP colored by the electrostatic potential (see the scale bar) calculated with APBS [68]. Low-affinity binding site is located around CcP residue D148, indicated by the arrow. Protein orientation is the same as in (A). (C), (E) Distributions of Cc centers of mass (CM) around CcP colored by the heme-W191 (C) or heme-heme (E) distances and the corresponding ET rates (see the scale bar). The left view is in the same orientation as in (A). The right view is obtained by rotation around the horizontal axis as indicated. Cc in the crystallographic orientation is shown as magenta cartoon. See Supplementary Videos S1 and S2 for an expanded view of (C) and (E), respectively. (D), (F) Distributions of the intermolecular heme-W191 (D) or heme-heme (F) distances (top) and the corresponding ET rates (bottom). Red and black traces are the distributions for the binary and ternary complexes, respectively. Solid vertical lines indicate the heme-W191 (D) or heme-heme (F) distance in the X-ray structure of the complex (top) and the corresponding ET rates (bottom). Dashed lines denote the largest experimentally measured ET rate in the Cc-CcP complex [41]. All molecular graphics were prepared with PyMOL [69].

**Figure 4:**
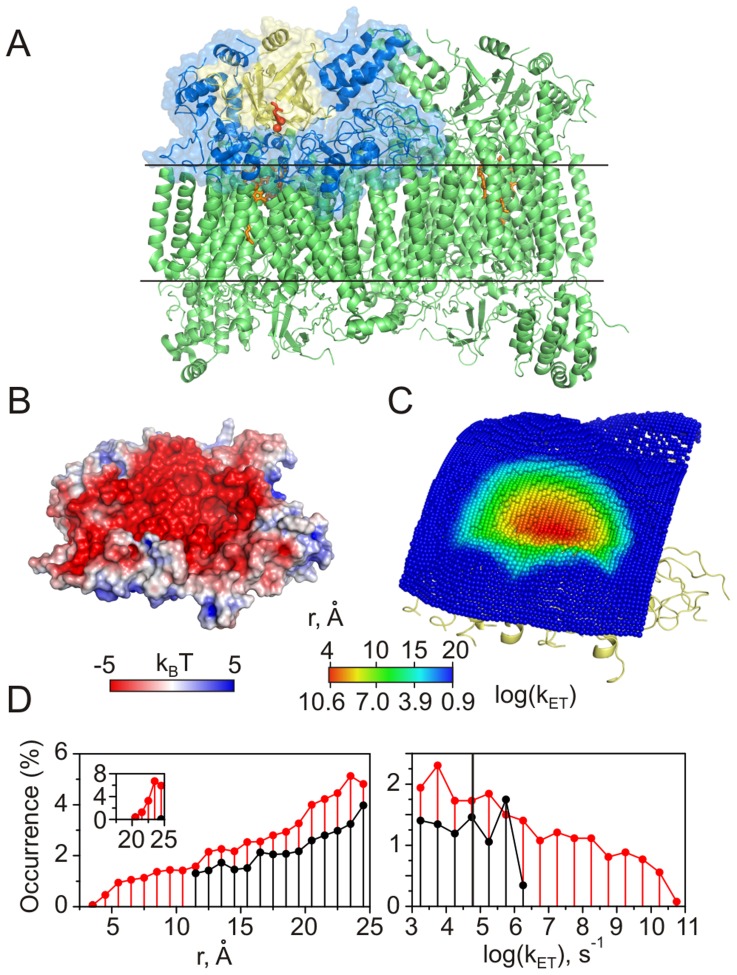
Cc-CCO. (A) Crystal structure of bovine CCO [70]. The intramitochondrial region of the CCO asymmetric unit modeled in this work is shown as a molecular surface. Subunit II is colored yellow, with its dinuclear CuA centre and W104 residue in red. The other CCO redox cofactors are shown in orange. Horizontal lines indicate approximate location of the mitochondrial membrane. (B) The molecular surface of the modeled CCO region coloured by the electrostatic potential (see the scale bar). Protein orientation is the same as in (A). (C) Distribution of Cc CMs around the subunit II colored by the heme-W104 distances and the corresponding ET rates (see the scale bar). Protein orientation is the same as in (A). (D) Distributions of the intermolecular distances (left) and ET rates (right) for heme-W104 (red) and heme-CuA (black). The solid vertical line indicates the fastest experimentally measured intermolecular ET rate in the Cc-CCO system [71]. The inset shows the distance distribution in the ternary complex (see text).

**Figure 5:**
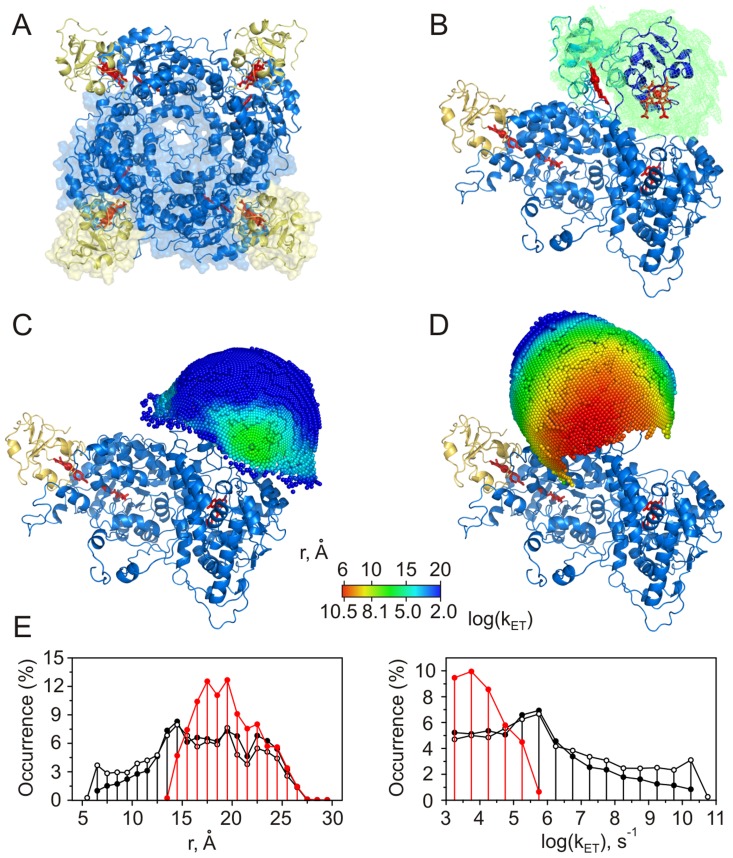
Cc-Fcb_2_. (A) Crystal structure of yeast Fcb_2_ [51]. Two of the four Cb_5_ heme domains (yellow), not observed in the crystal structure, were modeled in. The heme groups and FMN co-factors are colored red. The dimer modeled in this work is shown as a molecular surface. (B) Simulated ensemble of Fcb_2_ domain-domain orientations with residues 99–100 as a mobile linker. The green mesh is a reweighted atomic probability density map [72], plotted at a threshold of 10% maximum, for the overall distribution of the Cb_5_ domains among 100 generated structures. Two representative, low-energy solutions are shown in cyan and dark blue, with heme groups as red sticks. The crystallographic dimer is colored as in (A). (C)–(D) Distribution of Cc CMs around the Cb_5_ domain in (C) the crystallographic orientation or (D) a modeled domain-domain conformation (cyan structure in (B)), colored by the heme-heme distances and the corresponding ET rates (see the scale bar). (E) Distributions of the intermolecular heme-heme distances (left) and ET rates (right) for the crystallographic (red) and the simulated domain-domain orientations (black). Black open and filled symbols refer to the simulated ensemble members shown in (B) in cyan and dark blue, respectively.

**Figure 6:**
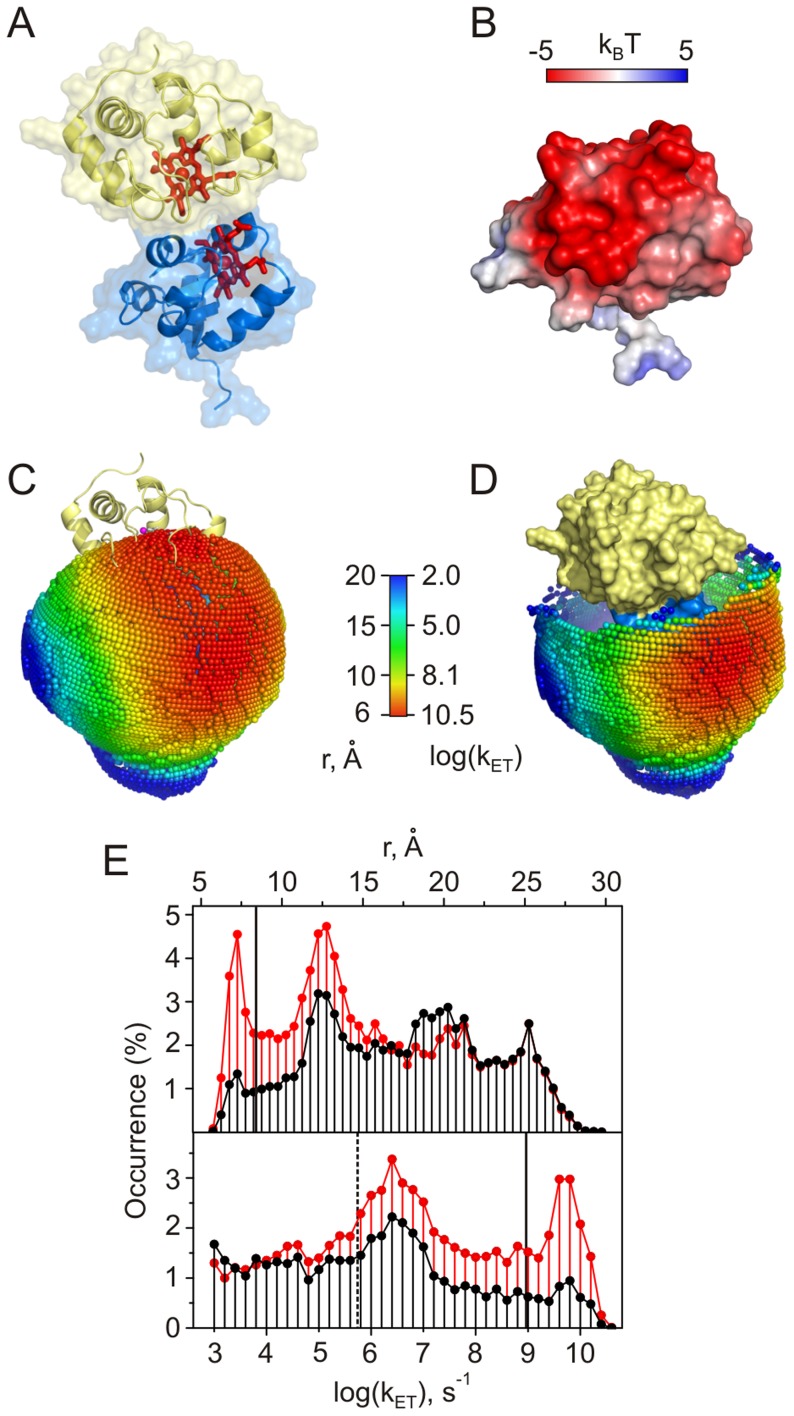
Cc-Cb_5_. (A) The hypothetical structural model of Salemme [5]. Cc and Cb_5_ are in yellow and blue, respectively, with heme groups shown as red sticks. (B) The molecular surface of Cb_5_ colored by the electrostatic potential (see the scale bar). Protein orientation is the same as in (A). (C), (D) Distribution of Cc CMs around Cb_5_ in the binary (C) and ternary (D) complexes colored by the heme-heme distances or the corresponding ET rates (see the scale bar). Protein orientation is the same as in (A). Cc in the Salemme model is shown as yellow cartoon (C) or molecular surface (D). See Videos S5 and S6 for an expanded view of (C) and (D), respectively. (E) Distributions of the intermolecular heme-heme distances (top) and ET rates (bottom). Red and black traces are the distributions for the binary and ternary complexes, respectively. Solid vertical lines indicate the heme-heme distance (top) and the corresponding ET rate (bottom) in the hypothetical model of Salemme [5]. The dashed line denotes the largest experimentally measured ET rate in the Cc-Cb_5_ complex [16].

The conformational search used in this work is not optimized for speed, but rather tailored for a fine sampling of the entire conformational space in the protein complexes. However, significant reduction in the computational time can be achieved by performing the rotational search and outputting the solutions only for the CMs with short separations between the ET cofactors (e.g. <20 Å). This in effect will discard most of the ET-inactive orientations (i.e. blue areas in the CM distribution maps in Figures 3–6, see below), while sampling the functional epitopes. A further gain in the calculation speed can be achieved by decreasing the resolution of the conformational search, either by increasing the rotational increments or decreasing the spatial resolution, δd. For instance, setting δχ and δψ to 15° instead of 5° and increasing the spacing between the neighboring CMs by 1 Å afford, respectively, 9- and 4-fold decrease in the number of sampled orientations. Combining the two strategies and restricting the search to the ET-competent areas only (see above) are expected to yield a 40- to 60-fold decrease in the computational time, depending on the system. Though the suggested scheme will miss out some of the ET-competent orientations (thus lowering the accuracy of the upper-limit k_ET_ estimates), it should still provide robust functional epitope maps.

### ET Rate Calculations

The ET rate constants can be estimated from the distances between redox centers as described by Dutton and co-workers [2], [35] or calculated from the ET pathway theory developed by Beratan and Onuchic [36]. The latter, computationally more demanding, approach requires the knowledge of the side-chain contacts across the interface, while the former relies solely on the separation between the electron donor and acceptor sites and appears to be more suitable to the rigid-body search method used here. In a broad range of biological systems, the ET rate constants are described by an exponential dependence on the distances between the redox centers (Equation 8 in the Methods section) [2]. Being a measure of the electronic coupling between the electron donor and acceptor, the exponential decay constant, β, is a property of the medium through which electron tunneling occurs, with the values varying from β = 1.0 Å^−1^ and β = 1.3 Å^−1^ for β-strands and α-helices, respectively, to β = 2.8 Å^−1^ for the vacuum [2], [3]. Analysis of biological ET systems showed that electron tunneling through a protein can be accurately described with a single value of β = 1.4 Å^−1^ [2], which is used throughout this work.

### Electron Transfer Complexes of Cc

#### Cc-CcP

Yeast CcP is a heme-containing enzyme catalyzing two-electron reduction of peroxides using reducing equivalents provided by Cc. Initial reaction with H_2_O_2_ yields compound I, containing Fe(IV) = O heme oxyferryl and a cationic indole radical W191^+•^, followed by a two-step reduction to generate the resting-state enzyme [21]. CcP possesses two Cc binding sites with markedly different binding affinities and ET properties. The crystal structure of Cc bound to the high-affinity site (Figure 3A) suggested an ET pathway leading from the Cc heme to the W191 group of CcP [37], while flash photolysis kinetics studies of Hoffman and co-workers [38], [39] demonstrated fast heme-heme ET from the Cc bound at the low-affinity site, presumably located in a negatively-charged CcP region identified in Brownian dynamics simulations (Figure 3B) [8]. The intermolecular ET in this complex has been studied in great detail; however, the relative contributions of the heme-W191 and heme-heme ET to the compound I reduction are still a matter of debate [21].

We used the Cc-CcP system to validate our computational approach and, in particular, verify whether it can predict the two ET-active sites. The results are presented in Figure 3. Panels (C) and (E) show the distributions of Cc centers of mass (CM) around CcP colored by the shortest heme-W191 (C) or heme-heme (E) distances determined from 7,776 rotamers at each Cc CM position (see the Conformational Sampling section above). The hotter the color, the smaller the distance and, consequently, the higher the intermolecular ET rate in that particular protein-protein orientation. A set of Cc CMs for the entire conformational space sampled determines the ET-competent epitope in the protein complex. The distributions of redox distances and the corresponding ET rates used for color-coding in panels (C) and (E) are given in plots (D) and (F).

Our analysis shows that protein-protein orientations exhibiting short heme-W191 distances and, consequently, fast heme-W191 ET rates are indeed located around the crystallographic, high-affinity binding site (Figure 3C,D). Moreover, most of these geometries are inaccessible in the ternary complex (compare red and black traces in Figure 3D; also Figure S1A), suggesting that the low-affinity site is incapable of sustaining fast heme-W191 ET. In contrast, the heme-heme ET-active orientations, found primarily in the low-affinity binding region, are virtually unaffected by Cc binding to the high-affinity site (Figures 3E,F and S1B). Thus, the present analysis confirms the findings of the experimental studies and correctly predicts the ET properties of the two binding sites.

When compared to the ET pathway coupling maps of Hoffman and co-workers [40], the heme-W191 functional epitope matches the CcP region exhibiting strong coupling, while the area supporting high heme-heme ET activity differs. This seeming discrepancy stems from the fact that the surface coupling maps, obtained for individual proteins, reflect the intrinsic ET properties of a given macromolecule [40], while the functional epitopes derived in this work report on the intermolecular ET in the protein complex.

Finally, we note that the experimentally measured heme-W191 ET rate is an order of magnitude higher than that calculated from the X-ray structure of the complex [41]. As the crystallographic binding orientation is preserved in solution [42], this discrepancy is not due to differences between the crystal and solution structures. Interestingly, a number of protein-protein orientations, located in the vicinity of the crystallographic binding site, exhibit shorter heme-W191 distances than that observed in the X-ray structure (Figure 3C,D), suggesting the possibility of a faster ET.

#### Cc-Cbc_1_ and Cc-CCO

Cbc_1_ and CCO are homodimeric, multisubunit, integral membrane protein complexes that mediate ET in the terminal branch of the mitochondrial respiratory chain, thereby generating proton gradient and membrane potential required for ATP synthesis. In the final step of the aerobic respiration, CCO channels the electron flow from Cc to O_2_ via a binuclear CuA centre, heme *a*, and heme *a*_3_/CuB site, the place of oxygen reduction [43]. The CuA center is located in the subunit II facing the mitochondrial intermembrane space (Figure 4A). Experimental work showed that neighboring residue W104 is critical for the rapid ET from Cc to CuA [44], [45] and may serve as an electron entry port [46]. At present, the structure of the Cc-CCO complex is not known. However, modeling studies suggest that Cc binds to a negatively-charged patch on the front side of subunit II, in close proximity to W104 [10], [47].

Cbc_1_ transfers electrons from ubiquinol to Cc via a Q-cycle mechanism involving four redox cofactors: two *b*-type hemes in the cytochrome *b* subunit, 2Fe2S iron-sulfur cluster in the Rieske protein, and the heme group in cytochrome *c*_1_ (Cc_1_) – the actual electron donor to Cc [48], [49]. The crystal structure of the Cc-Cbc_1_ complex from yeast shows a single Cc molecule bound to the Cbc_1_ dimer (Figure S3A). Recent molecular dynamics simulations suggest that the interaction between the Cc_1_ monomers triggers conformational changes, which alter the binding affinity for the second Cc molecule and lead to the observed asymmetric binding [11].

Simulating the interaction of Cc with the intramitochondrial part of the CCO monomer (blue surface in Figure 4A) reveals that the ET-competent binding geometries are located in a funnel-shaped area on the negatively-charged front face of subunit II (Figure 4B,C). We find a protein-protein orientation with the Cc heme and CCO W104 in vdW contact, enabling a remarkably fast ET. The edge-to-edge distance between the two redox centers (3.8 Å) is in a striking agreement with that reported for the best Cc-CCO docking solution in an earlier modeling study [10]. The direct ET from the Cc heme to the CCO CuA site is expected to be much slower due to the larger separation between the two groups (compare red and black traces in Figure 4D). The experimentally measured intermolecular ET rate is a million-fold slower than the upper k_ET_ limit estimated here (Table 1), suggesting that the ET occurs from a less active protein-protein orientation or is conformationally gated.

**Table 1:** ET properties of the Cc complexes modeled in this work.

Cc partner	Redox centre	r, Å^a^	k_ET_ upper limit^b^, 10^8^ s^−1^	ET epitope size^c^	k_ET_, 10^6^ s^−1^ measured^d^
CcP	W191	11.6	1.4 (0.36)	822	2 [41]
	heme	11.1	2.4 (0.81)	1276	
Cb_5_	heme	5.9	320 (113)	3306	0.55 [16]
Cbc_1_	Cc_1_ heme	6.2	0.15–2.7 (0.08–1.2)^e^	144–342^e^	0.014 [49]
CCO	W104	3.8	580 (131)	506	0.06 [71]
	CuA	11.3	0.017 (0.016)	18	
Erv1	C130–C133	4.5	20–350 (5–65)^e^	1341–2186^e^	
	FAD	4.6	5.6–100 (2.4–33)^e^	891–1622^e^	
Fcb2	X-ray	13.7	0.0067 (0)	0	
	Ensemble	5.7	470 (212)	2232±431^f^	
SOX	X-ray	6.1	170 (69)	2268	
	Ensemble	5.5	390 (101)	4665±916^f^	

a Shortest separation between redox cofactors;

b the values averaged over 10% of the most ET-active protein orientations with k_ET_>10^6^ s^−1^ are given in parentheses;

c number of Cc CMS with k_ET_>10^6^ s^−1^;

d experimentally measured (pseudo)first order intermolecular electron transfer rate constant (reference given in parentheses);

e the range of values reflects the uncertainty in the reorganization energy, λ (see Text S2);

f the average and the standard deviation calculated over all members of the simulated ensemble.

A steady-state kinetics study of CCO suggested the presence of two ET-active Cc binding sites with different affinities and catalytic properties [50], and an earlier modeling work identified a likely low-affinity binding region [10]. To assess the ET properties of the putative low-affinity site, we simulated the interaction of the second Cc molecule with the Cc-CCO binding geometry showing the shortest separation between the Cc heme and CCO W104 (see above). As most of the ET-competent protein-protein orientations are then effectively excluded by the steric interactions with the first Cc molecule, the resulting docking solutions show large separations between redox cofactors (Figure S2B), suggesting that the low-affinity binding site is not ET active.

We also modeled the interaction of Cc with the intramitochondrial part of the Cbc_1_ dimer (blue surface in Figure S3A). The Cc binding sites on the two Cbc_1_ monomers were treated as equivalent, and the data for a single, crystallographic site (Figure S3B) are reported. All ET-competent Cc-Cbc_1_ orientations are located in a well-defined patch around the crystallographic binding site, in a negatively-charged region of the Cbc_1_ surface (Figure S3C,D). As the reduction potentials of Cc and Cc_1_ hemes are nearly identical [49], i.e. activation energy ΔG^o^≈0, the Cc-Cbc_1_ system presents a good example of endergonic tunneling in biological ET. The experimentally measured intermolecular ET rate is 3–4 orders of magnitude smaller than our estimated k_ET_ limit (Table 1) and 20–350 times slower than the ET rate calculated from the X-ray structure (Figure S3E, left). Differences between the protein-protein orientations in crystal and solution or possible conformational gating of the ET have been advanced as possible explanations for the observed discrepancy [49].

#### Cc-Fcb_2_ and Cc-SOX

Yeast Fcb_2_ is a homotetrameric lactate dehydrogenase, with each subunit composed of a b_5_-like (Cb_5_) heme domain and a flavin mononucleotide (FMN)-binding domain, separated by a flexible linker. The FMN group undergoes a two-step reduction by the substrate lactate, with the electrons sequentially transferred to the Fcb_2_ heme group and Cc [23]. In the Fcb_2_ crystal structure [51], the two domains are in an orientation favorable for the intramolecular ET between the FMN and heme groups (Figure 5A,B). Interestingly, only two heme domains are observed in the tetramer, suggesting that the other pair is motionally disordered.

Found in animals, plants, and bacteria, SOX is a Mo-containing enzyme catalyzing sulfite oxidation in the final step of cysteine and methionine degradation [22]. The crystal structure of chicken SOX [52] shows a protein dimer, with each subunit consisting of a Cb_5_ heme domain and a Mo-binding domain, joined by a flexible tether (Figure S5A). Following the two-electron reduction of the Mo center by the substrate, the electrons are transferred to the SOX heme group, followed by the intermolecular ET to Cc [22]. In contrast to Fcb_2_, the crystallographic SOX orientation is not favorable for an interdomain ET.

In both Fcb_2_ and SOX, the domain-domain mobility is essential for the protein function [22], [23]. Albeit linked by a covalent tether, the two domains behave as a transient protein complex, with the heme domain alternating between the catalytic domain-bound and the Cc accessible states as exemplified by the X-ray structures of Fcb_2_ and SOX, respectively. Depending on the tether length and flexibility, the relative domain motions can sample a range of kinetic regimes spanning the “unimolecular” and “bimolecular” modes [53]. In order to assess the influence of the interdomain mobility on the intermolecular ET, we generated ensembles of SOX and Fcb_2_ domain-domain orientations and assessed their ET properties. For the crystallographic Fcb_2_ conformation, most of the Cc-Fcb_2_ binding geometries are not ET competent (Figure 5C and E, red trace). However, allowing flexibility of just two residues in the interdomain linker leads to an ensemble of structures with augmented solvent accessibility of the heme group (Figure 5B) and, consequently, markedly increased intermolecular ET rates (Figure 5D and E, black traces). With the shortest heme-heme distances of 13.7 Å in the crystallographic orientation and 5.7 Å in the modeled ensemble, such limited interdomain mobility affords five orders of magnitude enhancement in the ET upper limit (Table 1). Allowing higher degree of linker flexibility leads to more ET competent geometries, but does not further increase the k_ET_ value (Figure S4). Thus, a restricted interdomain motion pivoted on just two linker residues appears to be sufficient to enable fast intermolecular ET underlying the Fcb_2_ function.

In contrast to Fcb_2_, the crystallographic SOX heme domain orientation is favorable for the rapid ET to Cc (Figure S5C and E, red trace), with the majority of ET-competent docking solutions found in a negatively-charged interdomain cleft (Figure S5B). To simulate the largest extent of the domain-domain motion, we allowed full flexibility of the interdomain tether (residues 83–94) and generated an ensemble of heme domain orientations constrained only by the linker geometry and the vdW interactions with the other domain (Figure S5D). The shortest Cc-SOX heme-heme distances in the crystallographic orientation and the modeled ensemble are 6.1 Å and 5.5 Å, respectively, converting into very similar k_ET_ values (Table 1). Thus, though domain mobility leads to an increase in the number of ET-competent Cc binding geometries (Figure S5E, black trace), it does not change the upper limit k_ET_ value, suggesting that the relative domain orientation observed in the SOX crystal structure is sufficient for the rapid intermolecular ET necessary for the protein function.

#### Cc-Cb_5_ and Cc-Erv1

In what follows, we present the results for the Cc-Cb_5_ complex; the analysis of the Cc-Erv1 pair is given in the Text S3. Cb_5_ is a small hemoprotein carrying electrons between Cb_5_ reductase and a number of partners, including fatty acid desaturase, cytochrome P450 and methemoglobin [25]. Given the conflicting reports in the literature [20], [25], [26], it is not clear whether Cb_5_ interacts with Cc *in vivo*; however, *in vitro* the proteins form a complex, which has become a paradigm for the study of the intermolecular ET [54], [55]. At present, the Cc-Cb_5_ structure is not known. A hypothetical binding model of Salemme [5], featuring four charge-charge interactions across the interface and nearly coplanar heme groups (Figure 6A), is supported by ample experimental evidence [54], [55]. However, recent NMR studies revealed pronounced dynamics within the complex, suggesting the presence of multiple binding geometries [17], [56]. Depending on the source of Cb_5_ and the experimental conditions, formation of binary Cb_5_-Cc and ternary Cb_5_-(Cc)_2_ complexes has been reported [54]–[58].

In a binary complex, the ET-competent protein-protein orientations map out onto a broad patch of the Cb_5_ surface (Figure 6C), containing an area surrounding the Salemme binding site and comprising the binding geometries identified by Brownian dynamics [9] and experiment-driven docking [56]. Many of those orientations are still available in a ternary complex (Figure 6D), suggesting that Cc bound to the low-affinity Cb_5_ site can be highly ET-active (Figure 6E). The fastest experimentally measured ET rate is five orders of magnitude lower than the upper-limit k_ET_ value (Table 1) and *ca.* 1,700 times slower than that calculated for the Salemme structural model (Figure 6E, bottom panel). It was suggested before that the ET in this system might be “gated”, i.e. limited by protein dynamics within the complex [55]. If this is indeed the case, re-orientation of protein molecules to a more active bound form is expected to greatly decrease the overall observed ET rate, bringing it closer to the experimental value.

### ET Interactome of Cc

**Figure 7:**
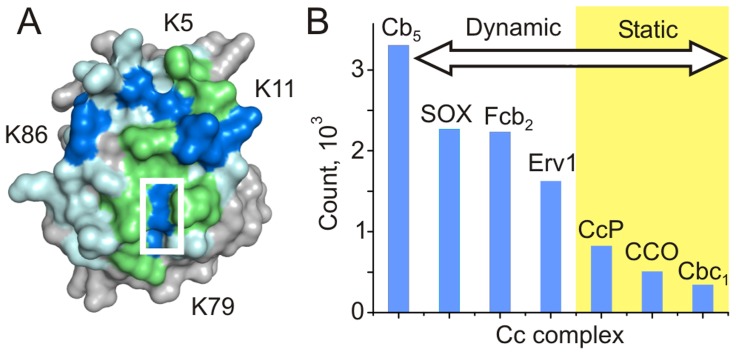
Cc binding interface and ET properties in redox complexes. (A) Combined binding interface of ferric yeast Cc (PDB entry 2YCC) [73] in complexes with CcP [74], Cb_5_ [56] and CCO [67] as determined by solution NMR spectroscopy. Residues experiencing binding effects in any of the three, two of the three, and all three complexes are colored light blue, green, and dark blue, respectively. The heme group is indicated by the white rectangle. Several lysine residues affected by the binding are labeled. (B) Absolute counts of Cc CMs with k_ET_>10^6^ s^−1^ in the ET complexes studied in this work.

The functional epitopes of the physiological Cc complexes derived in this work delineate the conformational space available to all ET-competent protein-protein orientations. For the complexes studied, the epitopes map onto the negatively-charged surfaces of the Cc partner proteins. As confirmed by the simulations where the Cc surface is sampled with the Cb_5_ and CcP molecules (Figures S6 and S7), Cc maintains frontal orientation in all ET-competent conformations, which is in excellent agreement with the experimental studies showing that the Cc binding interfaces in redox complexes comprise the surface-exposed heme edge and the surrounding ring of lysines (Figure 7A). Taken together, these findings highlight the importance of the electrostatic steering in the ET complexes and confirm that the exposed heme edge serves as the electron exit/entry port in Cc. It is noteworthy that the charge complementarity in Cc complexes is revealed by a method relying solely on steric properties of individual proteins. The fact that most protein-protein orientations with shortest separations between redox cofactors are also highly electrostatically favorable suggests that complementary electrostatics evolved to facilitate intermolecular ET.

The fact that the functional epitopes contain multiple, electrostatically favorable, ET-competent binding geometries suggests that in most cases the intermolecular ET can occur from more than one protein-protein orientation and might not require formation of a well-defined, stereospecific complex. Indeed, a recent experimental study of the interactions among a small copper protein amicyanin and several bacterial cytochromes has shown that a highly efficient ET can take place without formation of specific complexes, but rather via loose protein encounters guided by complementary electrostatics [59]. We hypothesize that the Cc ET interactome – particularly complexes with broad functional epitopes such as those in Cb_5_, Fcb_2_, SOX, and Erv1 – can exhibit a similar behavior.

It is well known that dynamics regulate the ET within proteins and, together with the fluctuations in the surrounding medium, enable ET gating [60]. Similarly, heterogeneity of the binding geometries in the ET complexes – arising from multiple protein-protein orientations sampled during the initial, encounter state of the biomolecular association [61] – is expected to modulate the intermolecular ET rates. Indeed, this notion has been gaining an increasing experimental support. For example, computational analysis of the kinetics data for the interaction of bacterial cytochrome c_2_ (Cc_2_) with the photosynthetic reaction centre (RC) revealed the presence of multiple ET-active binding geometries explored in the encounter state [62], [63]. In addition, detailed molecular dynamics simulations of the Cc_2_-RC encounter complex showed that strong ET coupling pathways between the two proteins include interstitial water molecules and illustrated that structural fluctuations within the encounter ensemble enable fast ET rates, in agreement with the experiment [63]. In another series of studies, Hoffman, Beratan and co-workers showed that interacting Cb_5_ and myoglobin sample multiple ET-active protein-protein orientations, constituting a subset of the binding geometries explored in the encounter state [19], [64]. Here, we use the functional epitope mapping to delineate the extent of the conformational space containing all ET-competent geometries, including those populated in the transient encounter complex.

The functional epitopes in Cc complexes differ in size and exhibit a large variation in the number of ET-competent binding geometries (Figure 7B). For example, a broad epitope in the Cc-Cb_5_ complex contains ten times more protein-protein orientations with k_ET_>10^6^ s^−1^ than a narrow, funnel-shaped ET patch in the Cc-Cbc_1_ complex. Interestingly, the size of the ET epitopes in Cc complexes correlates with the extent of protein dynamics. For instance, being a highly dynamic system consisting of multiple binding geometries [56], the Cc-Cb_5_ complex features a broad functional epitope, while the Cc-CcP complex – where proteins spend 70% of the time in the crystallographic binding orientation and 30% in a loose encounter state [32], [42] – has a much narrower ET-competent area. We suggest that the size of the ET epitope can be used as a diagnostic tool to discriminate between highly-dynamic, multiple-orientation complexes (e.g. Cc-Cb_5_, Cc-SOX, Cc-Fcb_2_) and their more static, predominantly single-orientation counterparts (e.g. Cc-CcP, Cc-CCO, and Cc-Cbc_1_) (Figure 7B). We hypothesize that differences in the widths of the ET-active areas reflect different evolutionary scenarios, contrasting the need to drive Cc binding to a well-defined, single orientation in Cbc_1_ and CCO complexes (served to prevent wasteful interactions with other parts of these multisubunit assemblies and the negatively-charged mitochondrial membrane) and allowing rapid ET from multiple protein-protein orientations in the Cc-Cb_5_ complex.

Without the knowledge of the populations of the protein-protein orientations constituting the functional epitope, the occurrences of donor-acceptor distances and the corresponding ET rates derived in this work cannot be converted into the ET probability distributions, and only the upper-limit k_ET_ values can be calculated. It is conceivable that some of the highly ET-competent binding geometries are energetically unfavorable and, therefore, do not form during the interaction. On the other hand, many thermodynamically favorable orientations (be they ET-active or not) might never get populated on the protein-protein association path. Thus, accurate predictions of the ET rates from the functional epitopes would require estimating the relative thermodynamic stabilities of the ET-competent binding geometries and addressing the protein association kinetics on a microscopic level. As illustrated by early modeling efforts of the Cc-CcP complex [6], failure to consider kinetic properties of a system led to an over-optimized predicted binding geometry (exhibiting high charge complementarity, large number of intermolecular hydrogen bonds, and snug steric fit of the protein sidechains), in stark contrast to the crystal structure featuring a single hydrogen bond and a loosely packed interface [37].

Used in the past to investigate Cc interactions with CcP [8], [65] and Cb_5_ [9], [54], Brownian dynamics (BD) simulations afford an effective weighting of various ET-competent orientations and allow extraction of bimolecular rate constants, k_on_, from association trajectories. When used with the ET reaction criteria (such as distances between the cofactors or the coupling strengths of ET pathways), the BD runs provide estimates for the intermolecular ET rates. Further, combination of BD and molecular dynamics (MD) allows to address the roles of structural fluctuations and the intervening solvent in the intermolecular ET process [63]. While BD and BD/MD are clearly methods of choice for accurate ET rate calculations, as a rule they require a complex force-field, adequate definition of the simulation conditions (e.g. proper partial charge and protonation state assignments for protein sidechains, boundary conditions, etc), a certain level of expertise, and considerable amount of time. Interestingly, the functional epitopes of Cc-CcP and Cc-Cb_5_ complexes delineated with our coarse-grained approach, relying solely on the steric properties of the interacting molecules, are in excellent agreement with those found in BD simulations [8], [9], [65]. Thus, it appears that the simple rigid-body conformational search employed in this work is sufficient for an adequate description of the ET-competent areas in Cc complexes.

As can be seen from the k_ET_ distributions, the studied Cc complexes contain a large number of binding geometries with the ET rates faster than the experimentally measured values. Even though not all of these ET-competent orientations will be populated in a given protein complex (see above), it seems likely that several highly-active conformations will dominate the intermolecular ET. This finding suggests that the overall ET rates in the Cc interactome are not limited by the ET event itself, but rather controlled by alterations in protein dynamics and/or conformational changes, occurring in protein complexes on slower time scales. Such binding-induced and/or redox-dependent conformational changes, reported for free Cc [66] and its complexes with Cbc_1_ [11] and CCO [67], could account for the apparent discrepancies between the observed and predicted ET rates.

### Concluding Remarks

The computational approach used here can be readily extended to other ET systems. Specifically, our analysis does not require the structures of protein complexes; can be applied in combination with homology modeling to systems with unknown protein structure(s) (as exemplified by Cc-Erv1); allows ensemble treatment of flexible groups (as shown for Cc complexes with Fcb_2_ and SOX); permits incorporation of side-chain optimization protocols (as done in control Cc-Cb_5_ runs); and enables calculations of the maximal, ‘activationless’ ET rates for the systems with unknown free energy changes (ΔG) and reorganization energies (λ). We believe that this work will engender future studies of the intermolecular ET in other biological networks.

## Supporting Information

Dataset S1 **Script files.**
(BZ2)

Figure S1 **Distributions of Cc CMs around CcP in the ternary complexes colored by the heme-W191 (A) or heme-heme (B) distances and the corresponding ET rates (see the scale bar).** Protein orientation is the same as in Figure 3A in the main text. Cc in the crystallographic orientation is shown as yellow surface. See Videos S3 and S4 for an expanded view of (A) and (B), respectively.
(TIF)

Figure S2 **Distribution of Cc CMs around the subunit II of CCO in binary (A) and ternary (B) complexes colored by the heme-CuA distances and the corresponding ET rates (see the scale bar).** Protein orientation is the same as in Figure 4C in the main text. (B) Cc bound to the high-affinity site of CCO is shown as a molecular surface.
(TIF)

Figure S3 **Cc-Cbc_1_.** (A) Crystal structure of the complex [S1]. The intramitochondrial region modeled in this work is shown as a blue molecular surface. Cc is colored yellow, heme groups of Cc and Cc_1_ are in red, the other Cbc_1_ redox cofactors are in orange. Horizontal lines indicate approximate location of the mitochondrial membrane. The antibody fragments used for protein crystallization are removed for clarity. (B) Close-up of the crystallographic Cc-Cbc_1_ orientation. (C) The molecular surface of the modeled Cbc_1_ region colored by the electrostatic potential (see the scale bar). Protein orientation is the same as in (B). (D) Distribution of Cc CMs around Cbc_1_ colored by the heme-heme distances and the corresponding ET rates (see the scale bar). Protein orientation is the same as in (B). (E) Distribution of the intermolecular heme-heme distances (left) and ET rates (right). In the right panel, the red and black traces indicate the ET rates calculated with λ = 0.7 and 1 ev, respectively. The solid vertical lines indicate the intermolecular heme-heme distance (left panel) and the corresponding ET rates (right panel; thick – calculated with λ = 0.7, thin – with λ = 1 ev) in the crystallographic orientation. The dashed line denotes the fastest experimentally measured intermolecular ET rate in the Cc-Cbc_1_ system [S2].
(TIF)

Figure S4 **Intermolecular ET in the Cc-Fcb_2_ complex with the simulated interdomain motion.** (A) Simulated ensemble of Fcb_2_ domain-domain orientations with residues 89–99 as a mobile linker. The green mesh is a reweighted atomic probability density map [S3], plotted at a threshold of 20% maximum, for the overall distribution of the Cb_5_ domains among 100 generated structures. Ten representative, low-energy solutions are shown as blue ribbons, with heme groups in red sticks. Crystallographic monomer is in cartoon. Protein orientation is the same as in Figure 5B in the main text. (B) Distributions of the intermolecular heme-heme distances (top) and ET rates (bottom) for the crystallographic (red) and the simulated domain-domain orientations (black). The latter was calculated for 10 solutions shown in (A), with the black line denoting the average.
(TIF)

Figure S5 **Cc-SOX.** (A) Crystal structure of chicken SOX [S4]. The monomer modeled in this work is shown as a molecular surface. Cb_5_ heme domain is colored yellow; heme groups and Mo atoms are in red. (B) The molecular surface of the SOX monomer colored by the electrostatic potential (see the scale bar). Protein orientation is the same as in (A). (C) Distribution of Cc CMs around the SOX heme *b*_5_ domain in the crystallographic orientation colored by the heme-heme distances and the corresponding ET rates (see the scale bar). Protein orientation is the same as in (A). (D) The green mesh is a reweighted atomic probability density map [S3], plotted at a threshold of 10% maximum, for the overall distribution of the SOX Cb_5_ domains among 100 generated structures. Fifteen representative, low-energy solutions are shown as blue ribbons, with heme groups in red sticks. Crystallographic monomer is in cartoon. (E) Distribution of the intermolecular heme-heme distances (left) and ET rates (right) for the crystallographic (red) and the simulated domain-domain orientations (black), the latter calculated for 15 solutions shown in (D), with the black line denoting the average.
(TIF)

Figure S6 **Functional epitope of Cc in the complex with Cb_5_.** (A) Cc and Cb_5_ in the hypothetical model of Salemme [S5] are in yellow and blue, respectively, with heme groups shown as sticks and iron atoms as red spheres. (B) Distribution of Cb_5_ CMs around Cc colored by the heme-heme distances (see the scale bar). Protein orientation is the same as in (A). See Video S7 for an expanded view.
(TIF)

Figure S7 **Functional epitope of Cc in the complex with CcP.** (A) Crystal structure of the complex [S6]. Cc and CcP are in yellow and blue, respectively, with heme groups shown as sticks and iron atoms as red spheres. (B) Distribution of CcP CMs around Cc colored by the heme-W191 distances (see the scale bar). Protein orientation is the same as in (A). See Video S8 for an expanded view.
(TIF)

Figure S8 **Cc-Erv1.** (A) Homology model of ScErv1 based on the crystal structure of AtErv1 [S7]. Redox-active groups are shown as red sticks. The isoalloxazine ring of FAD and the C130–C133 disulfide bridge are indicated by the labels. (B) Distribution of Cc CMs around ScErv1 colored by the flavin-heme (left) or disulfide-heme (right) distances and the corresponding ET rates (see the scale bar). Protein orientation is the same as in (A). (C) Distribution of the intermolecular distances (top) and ET rates (bottom) for flavin-heme (red traces) and disulfide-heme (black traces). The filled and open symbols in the bottom panel refer to the k_ET_ rates calculated with λ = 0.7 and 1 eV, respectively.
(TIF)

Figure S9 **The workflow in the computational protocol used for the Cc-CcP complex.** All scripts, input files, and some of the output data are provided in the Dataset S1.
(TIF)

Table S1 **Molecular systems modeled in this study.**
(PDF)

Text S1 **Description of the computational protocol.**
(PDF)

Text S2 **Details of the ET rate calculations.**
(PDF)

Text S3 **Cc-Erv1 complex.**
(PDF)

Text S4 **Supplementary references.**
(PDF)

Video S1 **Distribution of Cc CMs around CcP colored by the heme-W191 distances and the corresponding ET rates.** For details, see the legend to Figure 3 in the main text.
(MOV)

Video S2 **Distribution of Cc CMs around CcP colored by the heme-heme distances and the corresponding ET rates.** For details, see the legend to Figure 3 in the main text.
(MOV)

Video S3 **Distribution of Cc CMs around CcP in ternary complexes colored by the heme-W191 distances and the corresponding ET rates.** See the legend to Figure S1 for details.
(MOV)

Video S4 **Distribution of Cc CMs around CcP in ternary complexes colored by the heme-heme distances and the corresponding ET rates.** See the legend to Figure S1 for details.
(MOV)

Video S5 **Distribution of Cc CMs around Cb_5_ in binary complexes colored by the heme-heme distances and the corresponding ET rates.** For details, see the legend to Figure 6 in the main text.
(MOV)

Video S6 **Distribution of Cc CMs around Cb_5_ in ternary complexes colored by the heme-heme distances and the corresponding ET rates.** For details, see the legend to Figure 6 in the main text.
(MOV)

Video S7 **Functional epitope of Cc in the complex with Cb_5_.** Distribution of Cb_5_ CMs around Cc colored by the heme-heme distances. See the legend to Figure S6 for details.
(MOV)

Video S8 **Functional epitope of Cc in the complex with CcP.** Distribution of CcP CMs around Cc colored by the heme-W191 distances. See the legend to Figure S7 for details.
(MOV)

## References

[1] Marcus RA, Sutin N (1985) Electron transfers in chemistry and biology. Biochim Biophys Acta 811: 265–322.

[2] Moser CC, Keske JM, Warncke K, Farid RS, Dutton PL (1992) Nature of biological electron transfer. Nature 355: 796–802. 1311417 10.1038/355796a0

[3] Gray HB, Winkler JR (2005) Long-range electron transfer. Proc Natl Acad Sci USA 102: 3534–3539. 15738403 10.1073/pnas.0408029102 PMC553296

[4] Crowley PB, Ubbink M (2003) Close encounters of the transient kind: protein interactions in the photosynthetic redox chain investigated by NMR spectroscopy. Acc Chem Res 36: 723–730. 14567705 10.1021/ar0200955

[5] Salemme FR (1976) An hypothetical structure for an intermolecular electron transfer complex of cytochromes *c* and *b* _5_ . J Mol Biol 102: 563–568. 178879 10.1016/0022-2836(76)90334-x

[6] Poulos TL, Kraut J (1980) A hypothetical model of the cytochrome *c* peroxidase - cytochrome *c* electron transfer complex. J Biol Chem 255: 10322–10330. 6253470

[7] Wendoloski JJ, Matthew JB, Weber PC, Salemme FR (1987) Molecular dynamics of a cytochrome *c* - cytochrome *b* _5_ electron transfer complex. Science 238: 794–797. 2823387 10.1126/science.2823387

[8] Northrup SH, Boles JO, Reynolds JCL (1988) Brownian dynamics of cytochrome *c* and cytochrome *c* peroxidase association. Science 241: 67–70. 2838904 10.1126/science.2838904

[9] Northrup SH, Thomasson KA, Miller CM, Barker PD, Eltis LD, et al (1993) Effects of charged amino acid mutations on the bimolecular kinetics of reduction of yeast *iso*-1-ferricytochrome *c* by bovine ferrocytochrome *b* _5_ . Biochemistry 32: 6613–6623. 8392365 10.1021/bi00077a014

[10] Roberts VA, Pique ME (1999) Definition of the interaction domain for cytochrome *c* on cytochrome *c* oxidase. III. Prediction of the docked complex by a complete, systematic search. J Biol Chem 274: 38051–38060. 10608874 10.1074/jbc.274.53.38051

[11] Kokhan O, Wraight CA, Tajkhorshid E (2010) The binding interface of cytochrome *c* and cytochrome *c* _1_ in the *bc* _1_ complex: rationalizing the role of key residues. Biophys J 99: 2647–2656. 20959106 10.1016/j.bpj.2010.08.042 PMC2955499

[12] Gabdoulline RR, Wade RC (2001) Protein-protein association: investigation of factors influencing association rates by Brownian dynamics simulations. J Mol Biol 306: 1139–1155. 11237623 10.1006/jmbi.2000.4404

[13] Crowley PB, Carrondo MA (2004) The architecture of the binding site in redox protein complexes: implications for fast dissociation. Proteins 55: 603–612. 15103624 10.1002/prot.20043

[14] Erman JE, Kresheck GC, Vitello LB, Miller MA (1997) Cytochrome *c*/cytochrome *c* peroxidase complex: effect of binding-site mutations on the thermodynamics of complex formation. Biochemistry 36: 4054–4060. 9092837 10.1021/bi962632x

[15] Pielak GJ, Wang X (2001) Interactions between yeast *iso*-1-cytochrome *c* and its peroxidase. Biochemistry 40: 422–428. 11148036 10.1021/bi002124u

[16] Ren Y, Wang WH, Wang YH, Case M, Qian W, et al (2004) Mapping the electron transfer interface between cytochrome *b* _5_ and cytochrome *c* . Biochemistry 43: 3527–3536. 15035623 10.1021/bi036078k

[17] Prudêncio M, Ubbink M (2004) Transient complexes of redox proteins: structural and dynamic details from NMR studies. J Mol Recognit 17: 524–539. 15386621 10.1002/jmr.686

[18] Solmaz SRN, Hunte C (2008) Structure of complex III with bound cytochrome *c* in reduced state and definition of a minimal core interface for electron transfer. J Biol Chem 283: 17542–17549. 18390544 10.1074/jbc.M710126200

[19] Liang ZX, Nocek JM, Huang K, Hayes RT, Kurnikov IV, et al (2002) Dynamic docking and electron transfer between Zn-myoglobin and cytochrome *b* _5_ . J Am Chem Soc 124: 6849–6859. 12059205 10.1021/ja0127032

[20] Banci L, Assfalg M (2001) Mitochondrial cytochrome *c*. In: Messerschmidt A, Huber R, Poulos TL, Wieghardt K, editors. Handbook of Metalloproteins. Chichester: Wiley. pp 33–43.

[21] Volkov AN, Nicholls P, Worrall JAR (2011) The complex of cytochrome *c* and cytochrome *c* peroxidase: The end of the road? Biochim Biophys Acta 1807: 1482–1503. 21820401 10.1016/j.bbabio.2011.07.010

[22] Feng C, Tollin G, Enemark JH (2007) Sulfite oxidizing enzymes. Biochim Biophys Acta 1774: 527–539. 17459792 10.1016/j.bbapap.2007.03.006 PMC1993547

[23] Lederer F (2011) Another look at the interaction between mitochondrial cytochrome *c* and flavocytochrome *b* _2_ . Eur Biophys J 40: 1283–1299. 21503671 10.1007/s00249-011-0697-0

[24] Dabir DV, Leverich EP, Kim SK, Tsai FD, Hirasawa M, et al (2007) A role for cytochrome *c* and cytochrome *c* peroxidase in electron shuttling from Erv1. EMBO J 26: 4801–4811. 17972915 10.1038/sj.emboj.7601909 PMC2099471

[25] Mathews FS (2001) *b*-Type cytochrome electron carriers: cytochromes *b* _562_, *b* _5_, and flavocytochrome *b* _2_. In: Messerschmidt A, Huber R, Poulos TL, Wieghardt K, editors. Handbook of Metalloproteins. Chichester: Wiley. pp 159–171.

[26] Díaz-Moreno I, García-Heredia JM, Díaz-Quintana A, De la Rosa MA (2011) Cytochrome *c* signalosome in mitochondria. Eur Biophys J 40: 1301–1315. 22086608 10.1007/s00249-011-0774-4

[27] Kleywegt GJ, Jones TA (1998) Databases in protein crystallography. Acta Cryst D D54: 1119–1131. 10.1107/s0907444998007100 10089488

[28] Sali A, Blundell TL (1993) Comparative protein modelling by satisfaction of spatial restraints. J Mol Biol 234: 779–815. 8254673 10.1006/jmbi.1993.1626

[29] Vitu E, Bentzur M, Lisowsky T, Kaiser CA, Fass D (2006) Gain of function in an ERV/ALR sulfhydryl oxidase by molecular engineering of the shuttle disulfide. J Mol Biol 362: 89–101. 16893552 10.1016/j.jmb.2006.06.070

[30] Schwieters CD, Kuszewski JJ, Tjandra N, Clore GM (2003) The Xplor-NIH NMR molecular structure determination package. J Magn Reson 160: 66–74. 10.1016/s1090-7807(02)00014-9 12565051

[31] Iwahara J, Schwieters CD, Clore GM (2004) Ensemble approach for NMR structure refinement against ^1^H paramagnetic relaxation enhancement data arising from a flexible paramagnetic group attached to a macromolecule. J Am Chem Soc 126: 5879–5896. 15125681 10.1021/ja031580d

[32] Volkov AN, Ubbink M, van Nuland NAJ (2010) Mapping the encounter state of a transient protein complex by PRE NMR spectroscopy. J Biomol NMR 48: 225–236. 21049303 10.1007/s10858-010-9452-6 PMC3235994

[33] Brünger AT (1992) X-PLOR 3.1 manual. New Haven, CT: Yale University Press.

[34] Nilges M, Clore GM, Gronenborn AM (1988) Determination of three-dimensional structures of proteins from interproton distance data by hybrid distance geometry-dynamical simulated annealing calculations. FEBS Lett 229: 317–324. 3345845 10.1016/0014-5793(88)81148-7

[35] Page CC, Moser CC, Chen X, Dutton PL (1999) Natural engineering principles of electron tunnelling in biological oxidation-reduction. Nature 402: 47–52. 10573417 10.1038/46972

[36] Beratan DN, Betts JN, Onuchic JN (1992) Tunnelling pathway and redox-state-dependent electronic couplings at nearly fixed distance in electron-transfer proteins. J Phys Chem 96: 2852–2855.

[37] Pelletier H, Kraut J (1992) Crystal structure of a complex between electron transfer partners, cytochrome *c* peroxidase and cytochrome *c* . Science 258: 1748–1755. 1334573 10.1126/science.1334573

[38] Stemp EDA, Hoffman BM (1993) Cytochrome *c* peroxidase binds two molecules of cytochrome *c*: evidence for a low-affinity, electron-transfer-active site on cytochrome *c* peroxidase. Biochemistry 32: 10848–10865. 8399235 10.1021/bi00091a041

[39] Zhou JS, Hoffman BM (1994) Stern-Volmer in reverse: 2:1 stoichiometry of the cytochrome *c* - cytochrome *c* peroxidase electron-transfer complex. Science 265: 1693–1696. 8085152 10.1126/science.8085152

[40] Nocek JM, Zhou JS, de Forest S, Priyadarshy S, Beratan DN, et al (1996) Theory and practice of electron transfer within protein-protein complexes: application to multidomain binding of cytochrome *c* by cytochrome *c* peroxidase. Chem Rev 96: 2459–2490. 11848833 10.1021/cr9500444

[41] Wang K, Mei H, Geren L, Miller MA, Saunders A, et al (1996) Design of a ruthenium-cytochrome *c* derivative to measure electron transfer to the radical cation and oxyferryl heme in cytochrome *c* peroxidase. Biochemistry 35: 15107–15119. 8942678 10.1021/bi9611117

[42] Volkov AN, Worrall JAR, Holtzmann E, Ubbink M (2006) Solution structure and dynamics of the complex between cytochrome *c* and cytochrome *c* peroxidase determined by paramagnetic NMR. Proc Natl Acad Sci USA 103: 18945–18950. 17146057 10.1073/pnas.0603551103 PMC1748157

[43] Kaila VRI, Verkhovsky MI, Wikström M (2010) Proton-coupled electron transfer in cytochrome oxidase. Chem Rev 110: 7062–7081. 21053971 10.1021/cr1002003

[44] Zhen Y, Hoganson CW, Babcock GT, Ferguson-Miller S (1999) Definition of the interaction domain for cytochrome *c* on cytochrome *c* oxidase. I. Biochemical, spectral, and kinetic characterization of surface mutants in subunit II of *Rhodobacter Sphaeroides* cytochrome *aa* _3_ . J Biol Chem 274: 38032–38041. 10608872 10.1074/jbc.274.53.38032

[45] Wang K, Zhen Y, Sadoski R, Grinnell S, Geren L, et al (1999) Definition of the interaction domain for cytochrome *c* on cytochrome *c* oxidase. II. Rapid kinetic analysis of electron trasfer from cytochrome *c* to *Rhodobacter Sphaeroides* cytochrome oxidase surface mutants. J Biol Chem 274: 38042–38050. 10608873 10.1074/jbc.274.53.38042

[46] George SD, Metz M, Szilagyi RK, Wang H, Cramer SP, et al (2001) A quantitative description of the ground-state wave function of CuA by X-ray absorption spectroscopy: comparison to plastocyanin and relevance to electron transfer. J Am Chem Soc 123: 5757–5767. 11403610 10.1021/ja004109i

[47] Flöck D, Helms V (2002) Protein-protein docking of electron transfer complexes: cytochrome *c* oxidase and cytochrome *c* . Proteins 47: 75–85. 11870867 10.1002/prot.10066.abs

[48] Yu CA, Wen X, Xiao K, Xia D, Yu L (2002) Inter- and intra-molecular electron transfer in the cytochrome b*c* _1_ complex. Biochim Biophys Acta 1555: 65–70. 12206893 10.1016/s0005-2728(02)00256-6

[49] Engstrom G, Rajagukguk R, Sauders AJ, Patel CN, Rajagukguk S, et al (2003) Design of a ruthenium-labeled cytochrome *c* derivative to study electron transfer with the cytochrome *bc* _1_ complex. Biochemistry 42: 2816–2824. 12627947 10.1021/bi027213g

[50] Ferguson-Miller S, Brautigan DL, Margoliash E (1976) Correlation of the kinetics of electron transfer activity of various eukaryotic cytochromes *c* with binding to mitochondrial cytochrome *c* oxidase. J Biol Chem 251: 1104–1115. 2600

[51] Cunane LM, Barton JD, Chen ZW, Welsh FE, Chapman SK, et al (2002) Crystallographic study of the recombinant flavin-binding domain of baker's yeast flavocytochrome *b* _2_: comparison with the intact wild-type enzyme. Biochemistry 41: 4264–4272. 11914072

[52] Kisker C, Schindelin H, Pacheco A, Wehbi WA, Garrett RM, et al (1997) Molecular basis of sulfite oxidase deficiency from the structure of sulfite oxidase. Cell 91: 973–983. 9428520 10.1016/s0092-8674(00)80488-2

[53] Kawatsu T, Beratan DN (2006) Electron transfer between cofactors in protein domains linked by a flexible tether. Chem Phys 326: 259–269.

[54] Mauk AG, Mauk MR, Moore GR, Northrup SH (1995) Experimental and theoretical analysis of the interaction between cytochrome *c* and cytochrome *b* _5_ . J Bioenerg Biomemb 27: 311–330. 10.1007/BF02110101 8847345

[55] Durham B, Fairris JL, Mclean M, Millett F, Scott JR, et al (1995) Electron transfer from cytochrome *b* _5_ to cytochrome *c* . J Bioenerg Biomemb 27: 331–340. 10.1007/BF02110102 8847346

[56] Volkov AN, Ferrari D, Worrall JAR, Bonvin AMJJ, Ubbink M (2005) The orientations of cytochrome *c* in the highly dynamic complex with cytochrome *b* _5_ visualized by NMR and docking using HADDOCK. Prot Sci 14: 799–811. 10.1110/ps.041150205 PMC2279274 15689516

[57] Whitford D, Concar DW, Veitch NC, Williams RJP (1990) The formation of protein complexes between ferricytochrome *b* _5_ and ferricytochrome *c* studied using high-resolution ^1^H-NMR spectroscopy. Eur J Biochem 192: 715–721. 2170130 10.1111/j.1432-1033.1990.tb19281.x

[58] Banci L, Bertini I, Felli IC, Krippahl L, Kubicek K, et al (2003) A further investigation of the cytochrome *b* _5_ - cytochrome *c* complex. J Biol Inorg Chem 8: 777–786. 12884088 10.1007/s00775-003-0479-y

[59] Meschi F, Wiertz F, Klauss L, Blok A, Ludwig B, et al (2011) Efficient electron transfer in a protein network lacking specific interactions. J Am Chem Soc 133: 16861–16867. 21916462 10.1021/ja205043f

[60] Beratan DN, Skourtis SS, Balabin IA, Balaeff A, Keinan S, et al (2009) Steering electrons on moving pathways. Acc Chem Res 42: 1669–1678. 19645446 10.1021/ar900123t PMC2764794

[61] Ubbink M (2009) The courtship of proteins: understanding the encounter complex. FEBS Lett 583: 1060–1066. 19275897 10.1016/j.febslet.2009.02.046

[62] Miyashita O, Onuchic JN, Okamura MY (2004) Transition state and encounter complex for fast association of cytochrome *c* _2_ with bacterial reaction center. Proc Natl Acad Sci USA 101: 16174–16179. 15520377 10.1073/pnas.0405745101 PMC528947

[63] Miyashita O, Okamura MY, Onuchic JN (2005) Interprotein electron transfer from cytochrome *c* _2_ to photosynthetic reaction center: tunneling across an aqueous interface. Proc Natl Acad Sci USA 102: 3558–3563. 15738426 10.1073/pnas.0409600102 PMC553326

[64] Liang ZX, Kurnikov IV, Nocek JM, Mauk AG, Beratan DN, et al (2004) Dynamic docking and electron-transfer between cytochrome *b* _5_ and a suite of myoglobin surface-charged mutants. Introduction of a functional-docking algorithm for protein-protein complexes. J Am Chem Soc 126: 2785–2798. 14995196 10.1021/ja038163l

[65] Northrup SH, Luton JA, Boles JO, Reynolds JCL (1987) Brownian dynamics simulation of protein association. J Comput Aided Mol Des 1: 291–311. 10.1007/BF01677278 2848101

[66] Volkov AN, Vanwetswinkel S, Van de Water K, van Nuland NAJ (2012) Redox-dependent conformational changes in eukaryotic cytochromes revealed by paramagnetic NMR spectroscopy. J Biomol NMR 52: 245–256. 22318343 10.1007/s10858-012-9607-8

[67] Sakamoto K, Kamiya M, Imai M, Shinzawa-Itoh K, Uchida T, et al (2011) NMR basis for interprotein electron transfer gating between cytochrome *c* and cytochrome *c* oxidase. Proc Natl Acad Sci USA 108: 12271–12276. 21746907 10.1073/pnas.1108320108 PMC3145682

[68] Baker NA, Sept D, Joseph S, Holst MJ, McCammon JA (2001) Electrostatics of nanosystems: application to microtubules and the ribosome. Proc Natl Acad Sci USA 98: 10037–10041. 11517324 10.1073/pnas.181342398 PMC56910

[69] DeLano WL (2002) The PyMOL Molecular Graphics System. Palo Alto, CA: DeLano Scientific.

[70] Tsukihara T, Shimokata K, Katayama Y, Shimada H, Muramoto K, et al (2003) The low-spin heme of cytochrome *c* oxidase as the driving element of the proton-pumping process. Proc Natl Acad Sci USA 100: 15304–15309. 14673090 10.1073/pnas.2635097100 PMC307562

[71] Geren LM, Beasely JR, Fine BR, Saunders AJ, Hibdon S, et al (1995) Design of a ruthenium-cytochrome *c* derivative to measure electron transfer to the initial acceptor in cytochrome *c* oxidase. J Biol Chem 270: 2466–2472. 7852307 10.1074/jbc.270.6.2466

[72] Schwieters CD, Clore GM (2002) Reweighted atomic densities to represent ensembles of NMR structures. J Biomol NMR 23: 221–225. 12238594 10.1023/a:1019875223132

[73] Berghuis AM, Brayer GD (1992) Oxidation state-dependent conformational changes in cytochrome *c* . J Mol Biol 223: 959–976. 1311391 10.1016/0022-2836(92)90255-i

[74] Worrall JAR, Kolczak U, Canters GW, Ubbink M (2001) Interaction of yeast *iso*-1-cytochrome *c* with cytochrome *c* peroxidase investigated by [^15^N, ^1^H] heteronuclear NMR spectroscopy. Biochemistry 40: 7069–7076. 11401551 10.1021/bi0025823

