# Supplementary figures and images for: Electron Transfer Interactome of Cytochrome c
Source: PLoS Comput Biol. 2012 Dec 6;8(12):e1002807. doi: 10.1371/journal.pcbi.1002807 (PMC3516563; doi:10.1371/journal.pcbi.1002807)

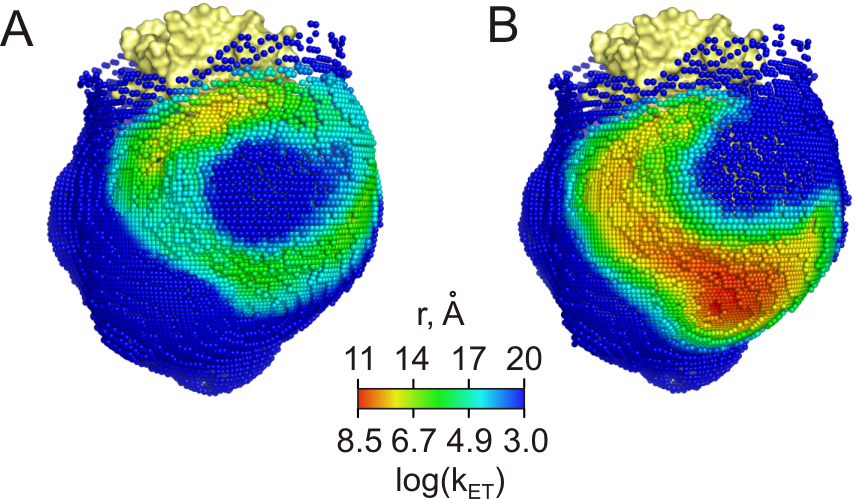

Supplement: Figure S1 — Distributions of Cc CMs around CcP in the ternary complexes colored by the heme-W191 (A) or heme-heme (B) distances and the corresponding ET rates (see the scale bar). Protein orientation is the same as in Figure 3A in the main text. Cc in the crystallographic orientation is shown as yellow surface. See Videos S3 and S4 for an expanded view of (A) and (B), respectively. (TIF) [file pcbi.1002807.s002.tif]

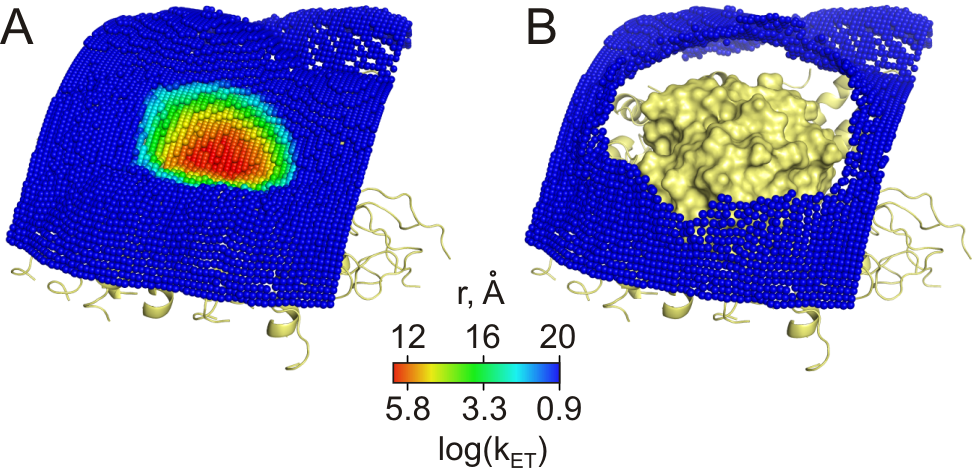

Supplement: Figure S2 — Distribution of Cc CMs around the subunit II of CCO in binary (A) and ternary (B) complexes colored by the heme-CuA distances and the corresponding ET rates (see the scale bar). Protein orientation is the same as in Figure 4C in the main text. (B) Cc bound to the high-affinity site of CCO is shown as a molecular surface. (TIF) [file pcbi.1002807.s003.tif]

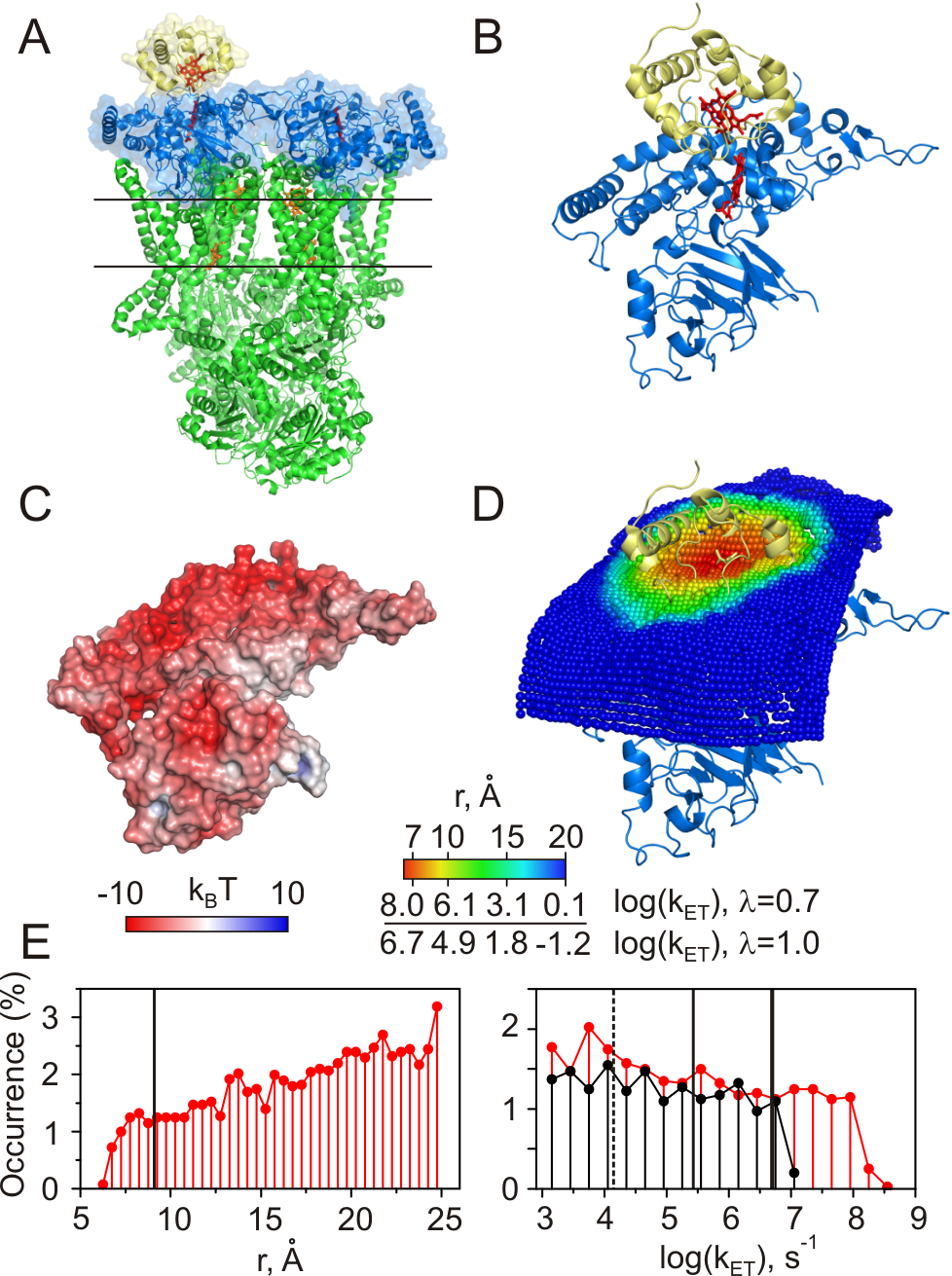

Supplement: Figure S3 — Cc-Cbc1. (A) Crystal structure of the complex [S1]. The intramitochondrial region modeled in this work is shown as a blue molecular surface. Cc is colored yellow, heme groups of Cc and Cc1 are in red, the other Cbc1 redox cofactors are in orange. Horizontal lines indicate approximate location of the mitochondrial membrane. The antibody fragments used for protein crystallization are removed for clarity. (B) Close-up of the crystallographic Cc-Cbc1 orientation. (C) The molecular surface of the modeled Cbc1 region colored by the electrostatic potential (see the scale bar). Protein orientation is the same as in (B). (D) Distribution of Cc CMs around Cbc1 colored by the heme-heme distances and the corresponding ET rates (see the scale bar). Protein orientation is the same as in (B). (E) Distribution of the intermolecular heme-heme distances (left) and ET rates (right). In the right panel, the red and black traces indicate the ET rates calculated with λ = 0.7 and 1 ev, respectively. The solid vertical lines indicate the intermolecular heme-heme distance (left panel) and the corresponding ET rates (right panel; thick – calculated with λ = 0.7, thin – with λ = 1 ev) in the crystallographic orientation. The dashed line denotes the fastest experimentally measured intermolecular ET rate in the Cc-Cbc1 system [S2]. (TIF) [file pcbi.1002807.s004.tif]

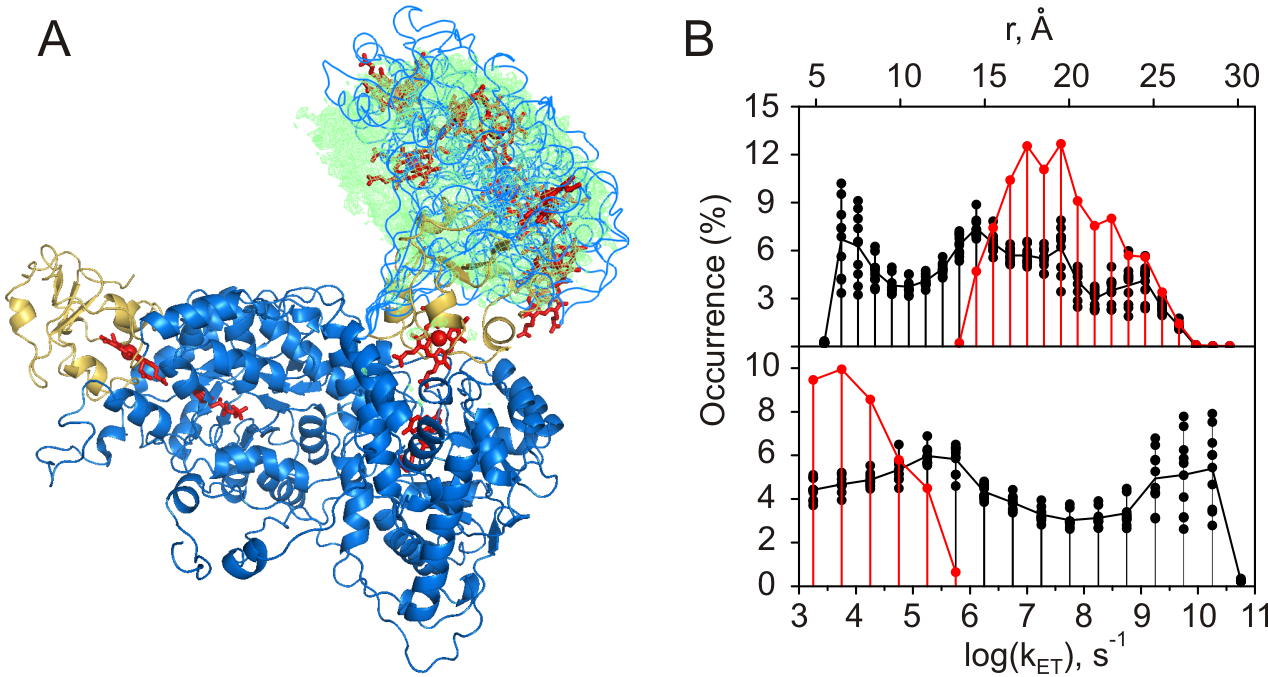

Supplement: Figure S4 — Intermolecular ET in the Cc-Fcb2 complex with the simulated interdomain motion. (A) Simulated ensemble of Fcb2 domain-domain orientations with residues 89–99 as a mobile linker. The green mesh is a reweighted atomic probability density map [S3], plotted at a threshold of 20% maximum, for the overall distribution of the Cb5 domains among 100 generated structures. Ten representative, low-energy solutions are shown as blue ribbons, with heme groups in red sticks. Crystallographic monomer is in cartoon. Protein orientation is the same as in Figure 5B in the main text. (B) Distributions of the intermolecular heme-heme distances (top) and ET rates (bottom) for the crystallographic (red) and the simulated domain-domain orientations (black). The latter was calculated for 10 solutions shown in (A), with the black line denoting the average. (TIF) [file pcbi.1002807.s005.tif]

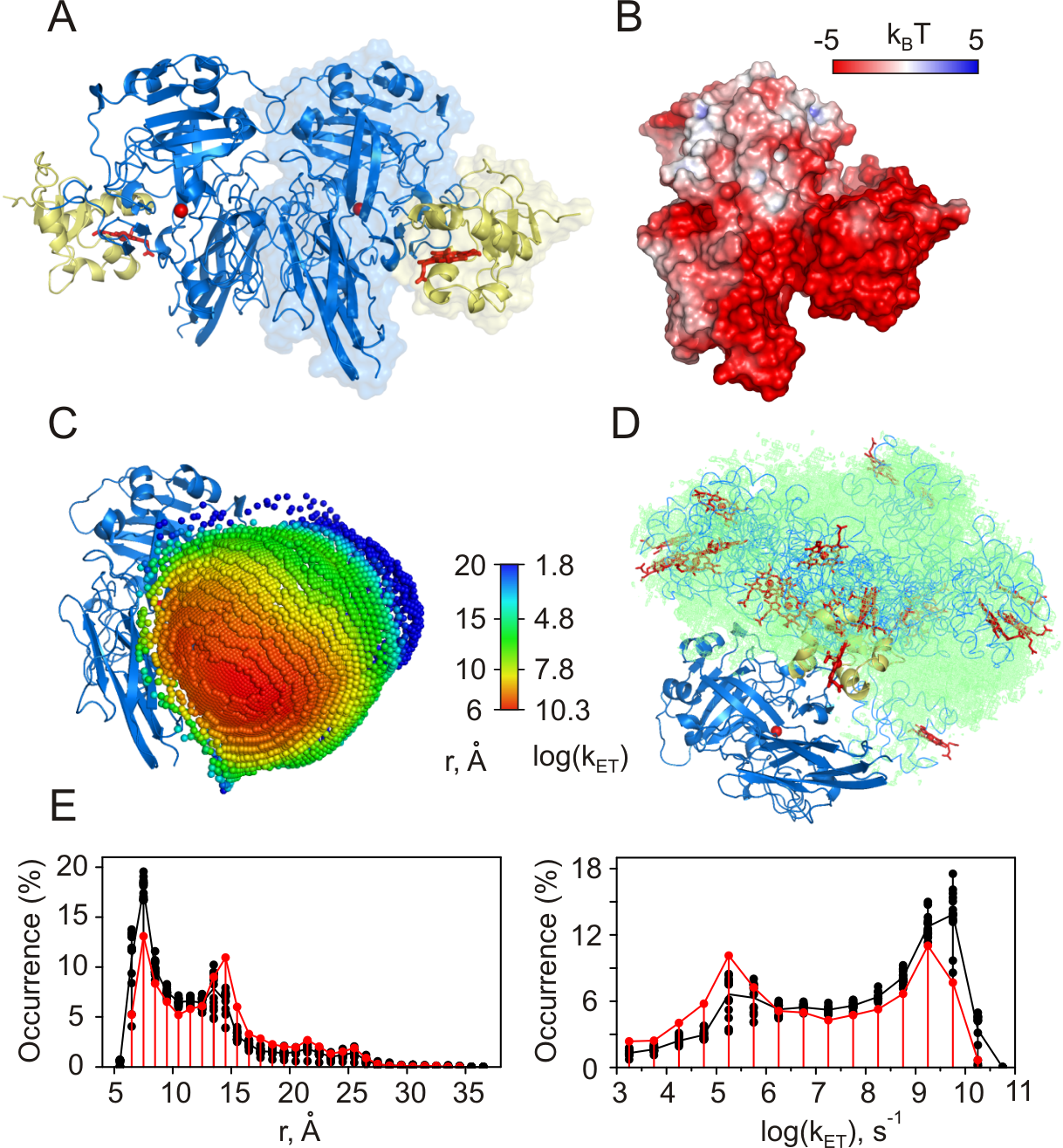

Supplement: Figure S5 — Cc-SOX. (A) Crystal structure of chicken SOX [S4]. The monomer modeled in this work is shown as a molecular surface. Cb5 heme domain is colored yellow; heme groups and Mo atoms are in red. (B) The molecular surface of the SOX monomer colored by the electrostatic potential (see the scale bar). Protein orientation is the same as in (A). (C) Distribution of Cc CMs around the SOX heme b 5 domain in the crystallographic orientation colored by the heme-heme distances and the corresponding ET rates (see the scale bar). Protein orientation is the same as in (A). (D) The green mesh is a reweighted atomic probability density map [S3], plotted at a threshold of 10% maximum, for the overall distribution of the SOX Cb5 domains among 100 generated structures. Fifteen representative, low-energy solutions are shown as blue ribbons, with heme groups in red sticks. Crystallographic monomer is in cartoon. (E) Distribution of the intermolecular heme-heme distances (left) and ET rates (right) for the crystallographic (red) and the simulated domain-domain orientations (black), the latter calculated for 15 solutions shown in (D), with the black line denoting the average. (TIF) [file pcbi.1002807.s006.tif]

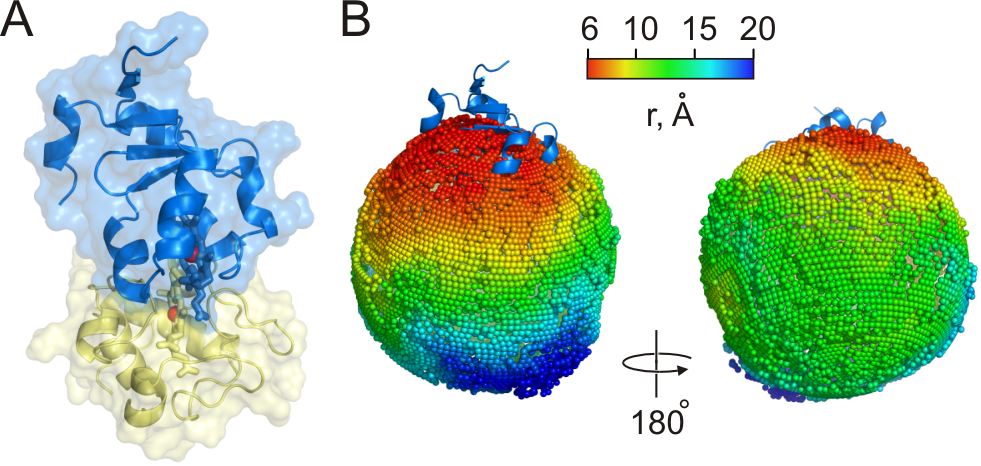

Supplement: Figure S6 — Functional epitope of Cc in the complex with Cb5. (A) Cc and Cb5 in the hypothetical model of Salemme [S5] are in yellow and blue, respectively, with heme groups shown as sticks and iron atoms as red spheres. (B) Distribution of Cb5 CMs around Cc colored by the heme-heme distances (see the scale bar). Protein orientation is the same as in (A). See Video S7 for an expanded view. (TIF) [file pcbi.1002807.s007.tif]

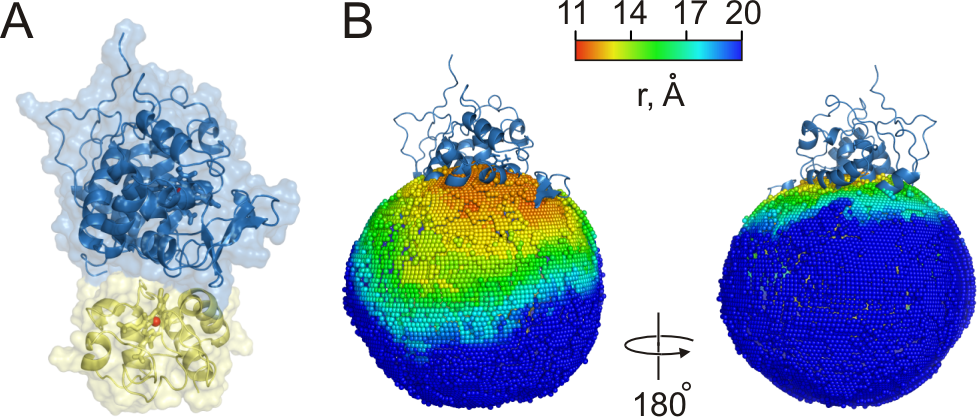

Supplement: Figure S7 — Functional epitope of Cc in the complex with CcP. (A) Crystal structure of the complex [S6]. Cc and CcP are in yellow and blue, respectively, with heme groups shown as sticks and iron atoms as red spheres. (B) Distribution of CcP CMs around Cc colored by the heme-W191 distances (see the scale bar). Protein orientation is the same as in (A). See Video S8 for an expanded view. (TIF) [file pcbi.1002807.s008.tif]

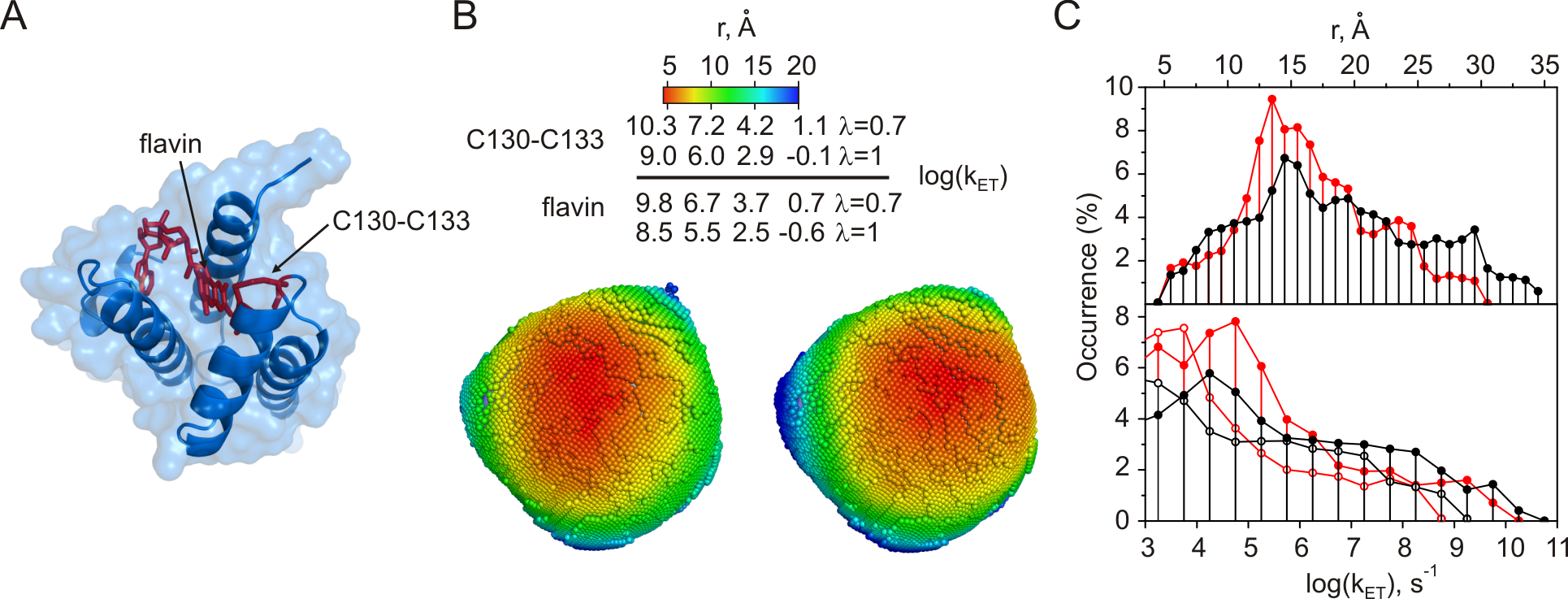

Supplement: Figure S8 — Cc-Erv1. (A) Homology model of ScErv1 based on the crystal structure of AtErv1 [S7]. Redox-active groups are shown as red sticks. The isoalloxazine ring of FAD and the C130–C133 disulfide bridge are indicated by the labels. (B) Distribution of Cc CMs around ScErv1 colored by the flavin-heme (left) or disulfide-heme (right) distances and the corresponding ET rates (see the scale bar). Protein orientation is the same as in (A). (C) Distribution of the intermolecular distances (top) and ET rates (bottom) for flavin-heme (red traces) and disulfide-heme (black traces). The filled and open symbols in the bottom panel refer to the kET rates calculated with λ = 0.7 and 1 eV, respectively. (TIF) [file pcbi.1002807.s009.tif]

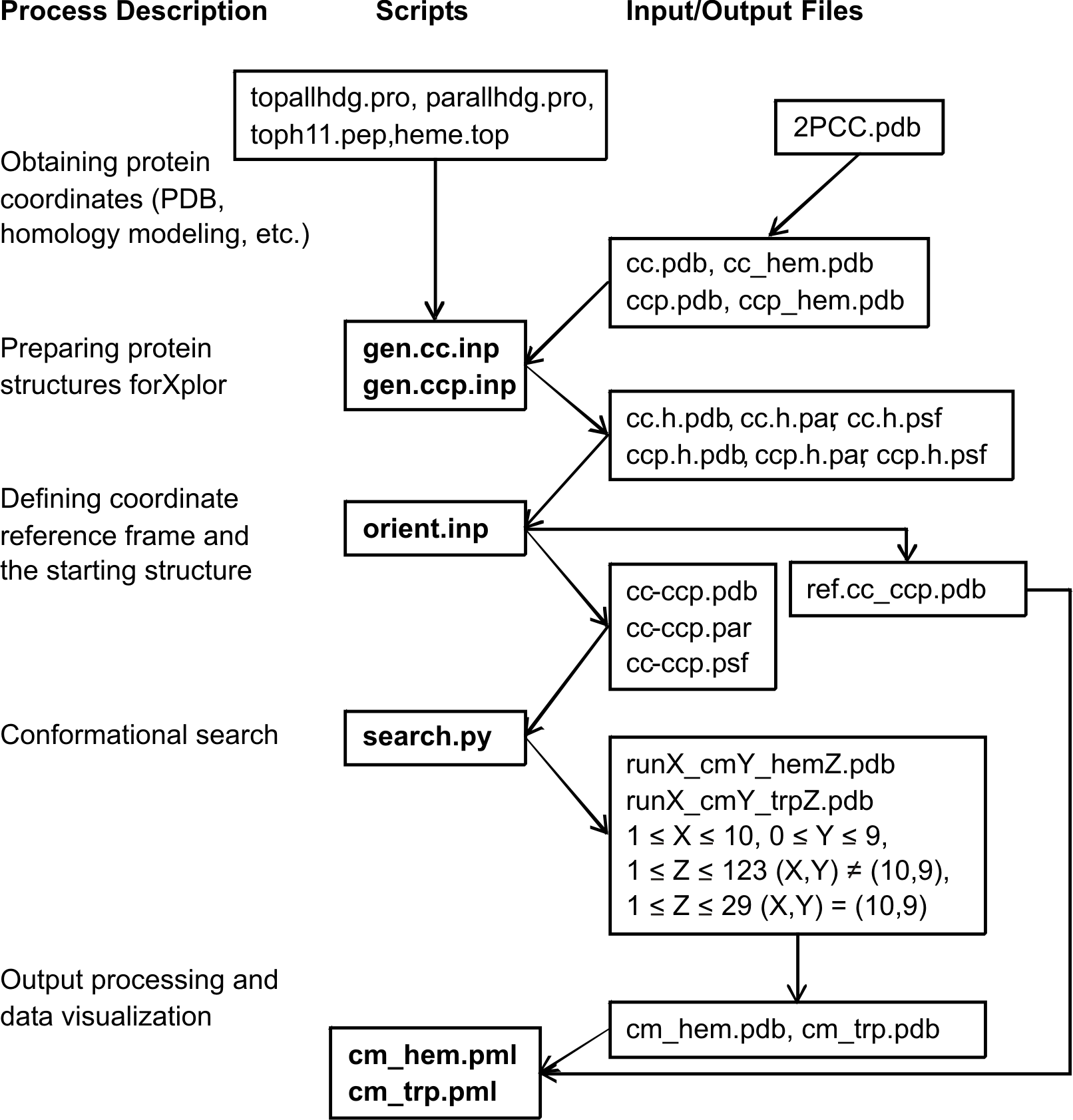

Supplement: Figure S9 — The workflow in the computational protocol used for the Cc-CcP complex. All scripts, input files, and some of the output data are provided in the Dataset S1. (TIF) [file pcbi.1002807.s010.tif]
