# Supplementary material for: Electron Transfer Interactome of Cytochrome c
Source: PLoS Comput Biol. 2012 Dec 6;8(12):e1002807. doi: 10.1371/journal.pcbi.1002807 (PMC3516563; doi:10.1371/journal.pcbi.1002807)
Supplement: Table S1 — Molecular systems modeled in this study. (PDF) [file pcbi.1002807.s011.pdf]

Stationary protein[a]	Moving protein	Atoms[b]	Structures[c]
yeast CcP (2PCC) [S6]	yeast Cc (2PCC) [S6]	6,475	94,913,856
bovine Cb_5 (1CYO) [S22]	yeast Cc (1YCC) [S23]	3,255	59,797,440
yeast Cbc_1 (3CX5) [S1] chains D (62-261); E (91-215); F; I (46-58); O (62-261); P (91-215); Q; T (46-58)	yeast Cc (1YCC) [S23]	14,629	35,435,232
bovine CCO (1V54) [S24] chains A (37-55, 115-144, 209-232, 287-302, 361-374, 428-451); B (2-20, 85-228); C (31-43, 99-127, 184-196, 255-261); D (99-147); G (41-84); H; I (39-73); J (53-58); K (33-54); L (40-47); M (33-43)	horse Cc (1HRC) [S25]	11,442	40,481,856
yeast Fcb_2 (1KBI) [S26] chains A and B. Missing heme domain in chain B modeled based on that in chain A. Missing residues in the 300-308 loop modeled.	yeast Cc (1YCC) [S23]	18,064	38,677,824-54,820,800
chicken SOX (1SOX) [S4] chain A. Missing atoms in residues 85, 86, 89, and 94 modeled.	horse Cc (1HRC) [S25]	8,939	34,992,000-69,470,784
residues 84-183 of the yeast Erv1 homology-modeled on the structure of AtErv1 (2HJ3) [S7]	yeast Cc (1YCC) [S23]	3,482	85,769,280

[a] Residue selections for parts of modeled protein chains are given in parentheses; [b] total number of atoms in the system; [c] number of structures sampled per run.
