# Supplementary material for: Electron Transfer Interactome of Cytochrome c
Source: PLoS Comput Biol. 2012 Dec 6;8(12):e1002807. doi: 10.1371/journal.pcbi.1002807 (PMC3516563; doi:10.1371/journal.pcbi.1002807)
Supplement: Text S1 — Description of the computational protocol. (PDF) [file pcbi.1002807.s012.pdf]

The computational protocol was implemented as a pipeline of several Xplor-NIH scripts, where output of one script is the input for the next. A typical workflow is illustrated on the example of the Cc-CcP complex in Figure S9. See below for a brief description of the scripts.

gen.cc.inp, gen.ccp.inp

These scripts generate Xplor parameter and structure files for Cc and CcP and add missing hydrogen atoms according to the standard Xplor topology and parameter sets (topallhdg.pro, top11.pep, and parallhdg.pro). For the heme groups and their ligands we used the topology and parameter files available in our group; however, those can also be obtained from the HIC-Up database (http://xray.bmc.uu.se/hicup/). The Cc and CcP coordinates were taken from the PDB entry for the Cc-CcP complex (2PCC). Coordinates for two protein chains and cofactors were placed in separate files and used as input.

orient.inp

This script combines Cc and CcP into a single molecular structure; places pseudoatoms (atom name G, residue name GB) at the proteins' CMs; orients the protein complex in the reference frame defined in Figure 1A (main text) so that CcP and Cc CMs appear at the coordinate origin and on the positive z axis, respectively; and outputs the reference coordinate file (ref.cc-ccp.pdb). Further, the script finds the protein-protein orientation with the minimal Cc-CcP heme-heme separation at the original, fixed CM positions (i.e. the structure with the frontal Cc orientation, Figure 1B in the main text), which is used as an input for the subsequent conformational search.

search.py

The script performs a complete conformational search of the Cc-CcP binding geometries and outputs the coordinates of the Cc CMs, which define the coverage of the space sampled. As a rule, the entire search was split among 10 runs, using 10 CPUs each. The current run number and the total number of CPUs used to carry out the search (given by the variables *run_number* and *nproc_tot*, respectively) define the number of (θ, φ) increments processed in each job. For each (θ, φ) increment, the script outputs the coordinates of the Cc CMs with the smallest Cc-CcP heme-heme (runX_cmY_hemZ.pdb) and heme-W191 (runX_cmY_trpZ.pdb) distances as the b-factors. X, Y, and Z are the respective run, job, and output structure numbers. Translational (θ, φ) and rotational (χ, ψ, ξ) coordinates for each Cc CM are given in the captions of the output files. First 10 output files are given for bench-marking purposes.

cm_hem.pml, cm_trp.pml

Pymol scripts for data visualization. See Figure 3C,E in the main text for details.
