# Supplementary material for: Electron Transfer Interactome of Cytochrome c
Source: PLoS Comput Biol. 2012 Dec 6;8(12):e1002807. doi: 10.1371/journal.pcbi.1002807 (PMC3516563; doi:10.1371/journal.pcbi.1002807)
Supplement: Text S2 — Details of the ET rate calculations. (PDF) [file pcbi.1002807.s013.pdf]

The ET rates were calculated from Equation 8 in the main text using the ΔG^o and λ values given below.

Cc-CcP. For the ET from yeast Cc to the CcP W191$^{+\bullet}$ radical, the driving force matches the reorganization energy ($-\Delta G^o \approx \lambda$), so that the ET is described by the maximum, activationless rate constant $k_{ET} \approx k_{ET,max}$ [S8]. For the ET to the CcP heme oxyferryl, λ = 0.7 eV [S9] and ΔG^o = -797 mV can be calculated from the reduction potentials (ΔE) of yeast Cc, ΔE = 290 mV [S10], and the CcP oxyferryl, ΔE = 1087 mV [S11].

Cc-Cb$_5$. For the heme-heme ET between bovine Cb$_5$ and yeast Cc, λ = 0.7 eV [S12] and ΔG^o = -270 mV is calculated from ΔE = 20 mV for Cb$_5$ [S13] and ΔE = 290 mV for Cc [S10].

Cc-Cbc$_1$. The reduction potentials of yeast Cc and Cc$_1$ hemes are nearly identical (i.e. $\Delta G^o \approx 0$) [S2], while the reorganization energy is estimated to be in the range λ = 0.7 - 1 eV [S2].

Cc-CCO. With ΔE = 245 mV for the CuA site [S14] and ΔE = 220 mV for the horse Cc bound to CCO [S15], ΔG^o = -25 mV. The reorganization energy for the intermolecular ET step, λ, can be calculated from the reorganization energies of Cc, $\lambda_1 \approx 0.7$ eV [S16], and the CuA site, $\lambda_2 \approx 0.4$ eV [S17], as $\lambda = (\lambda_1 + \lambda_2)/2 = 0.55$ eV.

Cc-Fcb$_2$. With ΔE = 6 mV for the heme b$_5$ of Fcb$_2$ [S18] and ΔE = 290 mV for the yeast Cc [S10], ΔG^o = -284 mV; λ = 0.7 eV as reported for the Cc-Cb$_5$ couple [S12].

Cc-SOX. With ΔE = 20 mV for the heme group in Cb$_5$ [S13] and ΔE = 260 mV for the horse Cc [S19], ΔG^o = -240 mV; λ = 0.7 eV [S12].

Cc-Erv1. Given the values of ΔE = 290 mV for the yeast Cc [S10], ΔE = 215 mV for the Erv1 flavin [S20], and ΔE = 150 mV for the C130-C133 disulfide in Erv1 [S20], the activation energy for the ET from the Erv1 flavin or C130-C133 disulfide is ΔG^o = -75 mV or -140 mV, respectively. A range of reorganization energies (λ = 0.7 - 1 eV) was used in the calculations.
