# Supplementary material for: Electron Transfer Interactome of Cytochrome c
Source: PLoS Comput Biol. 2012 Dec 6;8(12):e1002807. doi: 10.1371/journal.pcbi.1002807 (PMC3516563; doi:10.1371/journal.pcbi.1002807)
Supplement: Text S3 — Cc-Erv1 complex. (PDF) [file pcbi.1002807.s014.pdf]

Erv1 is a flavin-dependent sulfhydryl oxidase that aides the import of cysteine-rich proteins in the mitochondrial intermembrane space [S20]. It accepts electrons from Mia40 – an enzyme catalyzing formation of disulfide bonds – and passes them to Cc, acting as the electron sink. Erv1 contains three redox-active centers: two disulfides, C30-C33 (distal) and C130-C133 (proximal), and a flavin adenine nucleotide (FAD). The distal cysteine pair, believed to mediate Erv1 homodimerization, was shown to be the electron entry site in ALR, a flavin-dependent sulfhydryl oxidase in humans [S21]. In ALR, the electrons are further relayed to the proximal disulfide and then the FAD group, the electron donor to Cc [S21]. A similar electron flow is expected in Erv1; however, direct ET from the proximal disulfide to Cc was also suggested [S20]. In order to investigate the ET properties of the two redox sites, we modeled the interaction of Cc with the *Saccharomyces cerevisiae* Erv1 (ScErv1).

As the ScErv1 structure is not known, we built a structural model based on the X-ray coordinates of a homologous protein from *Arabidopsis thaliana* (AtErv1). Given that the first 80 residues, constituting a flexible N-terminal segment involved in protein dimerization and interaction with Mia40, are not observed in the AtErv1 structure, the ScErv1 model was built for residues 84-183 containing the proximal disulfide and the FAD group (Figure S8A). The ET-competent Cc-Erv1 orientations are located in well-defined patches on the front face of the protein (Figure S8B), with a number of protein-protein geometries featuring short ET distances (Figure S8C). Thus, from the geometrical point of view, it appears that both the proximal disulfide and the FAD group of ScErv1 can sustain fast ET to Cc.
