# Supplementary material for: Electron Transfer Interactome of Cytochrome c
Source: PLoS Comput Biol. 2012 Dec 6;8(12):e1002807. doi: 10.1371/journal.pcbi.1002807 (PMC3516563; doi:10.1371/journal.pcbi.1002807)
Supplement: Text S4 — Supplementary references. (PDF) [file pcbi.1002807.s015.pdf]

S1. Solmaz SRN, Hunte C (2008) Structure of complex III with bound cytochrome *c* in reduced state and definition of a minimal core interface for electron transfer. *J Biol Chem* 283: 17542-17549.

S2. Engstrom G, Rajagukguk R, Sauders AJ, Patel CN, Rajagukguk S, et al. (2003) Design of a ruthenium-labeled cytochrome *c* derivative to study electron transfer with the cytochrome bc_1 complex. *Biochemistry* 42: 2816-2824.

S3. Schwieters CD, Clore GM (2002) Reweighted atomic densities to represent ensembles of NMR structures. *J Biomol NMR* 23: 221-225.

S4. Kisker C, Schindelin H, Pacheco A, Wehbi WA, Garrett RM, et al. (1997) Molecular basis of sulfite oxidase deficiency from the structure of sulfite oxidase. *Cell* 91: 973-983.

S5. Salemme FR (1976) An hypothetical structure for an intermolecular electron transfer complex of cytochromes *c* and b_5. *J Mol Biol* 102: 563-568.

S6. Pelletier H, Kraut J (1992) Crystal structure of a complex between electron transfer partners, cytochrome *c* peroxidase and cytochrome *c*. *Science* 258: 1748-1755.

S7. Vitu E, Bentzur M, Lisowsky T, Kaiser CA, Fass D (2006) Gain of function in an ERV/ALR sulfhydryl oxidase by molecular engineering of the shuttle disulfide. *J Mol Biol* 362: 89-101.

S8. Wang K, Mei H, Geren L, Miller MA, Saunders A, et al. (1996) Design of a ruthenium-cytochrome *c* derivative to measure electron transfer to the radical cation and oxyferryl heme in cytochrome *c* peroxidase. *Biochemistry* 35: 15107-15119.

S9. Summers FE, Erman JE (1988) Reduction of cytochrome *c* peroxidase compounds I and II by ferrocytochrome *c*. A stopped-flow kinetic investigation. *J Biol Chem* 263: 14267-14275.

S10. Barker PD, Mauk AG (1992) pH-linked conformational regulation of a metalloprotein oxidation-reduction equilibrium: electrochemical analysis of the alkaline form of cytochrome *c*. *J Am Chem Soc* 114: 3619-3624.

S11. Purcell WL, Erman JE (1976) Cytochrome *c* peroxidase catalyzed oxidations of substitution inert iron(II) complexes. *J Am Chem Soc* 98: 7033-7037.

S12. McLendon G, Miller JR (1985) The dependence of biological electron transfer rates on exothermicity. The cytochrome *c*/cytochrome b_5 couple. *J Am Chem Soc* 107: 7811-7816.

S13. Velick SF, Strittmatter P (1956) The oxidation-reduction stoichiometry and potential of microsomal cytochrome. *J Biol Chem* 221: 265-275.

S14. Erecinska M, Chance B, Wilson DK (1971) The oxidation-reduction potential of the copper signal in pigeon heart mitochondria. *FEBS Lett* 16: 284-286.

S15. Schroedl NA, Hartzell CR (1977) Oxidative titrations of reduced cytochrome aa_3: correlation of midpoint potentials and extinction coefficients observed at three major absorption bands. *Biochemistry* 16: 4961-4965.

S16. Sharp KA (1998) Calculation of electron transfer reorganization energies using the finite difference Poisson-Boltzmann model. *Biophys J* 73: 1241-1250.

S17. Farver O, Lu Y, Ang MC, Pecht I (1999) Enhanced rate of intramolecular electron transfer in an engineered purple CuA azurin. *Proc Natl Acad Sci USA* 96: 899-902.

S18. Capeillère-Blandin C, Barber MJ, Bray RC (1986) Comparison of the processes involved in reduction by the substrate for two homologous flavocytochromes b_2 from different species of yeast. *Biochem J* 238: 745-756.

S19. Banci L, Assfalg M (2001) Mitochondrial cytochrome *c*. In: Messerschmidt A, Huber R, Poulos TL, Wieghardt K, editors. *Handbook of Metalloproteins*. Chichester: Wiley, pp. 33-43.

S20. Dabir DV, Leverich EP, Kim SK, Tsai FD, Hirasawa M, et al. (2007) A role for cytochrome *c* and cytochrome *c* peroxidase in electron shuttling from Erv1. *EMBO J* 26: 4801-4811.

S21. Banci L, Bertini I, Calderone V, Cefaro C, Ciofi-Baffoni S, et al. (2012) An electron-transfer path through an extended disulfide relay system: the case of the redox protein ALR. *J Am Chem Soc* 134: 1442-1445.

S22. Durley RC, Mathews FS (1996) Refinement and structural analysis of bovine cytochrome b_5 at 1.5 Å resolution. *Acta Crystallogr , Sect D: Biol Crystallogr* 52: 65-76.

S23. Louie GV, Brayer GD (1990) High-resolution refinement of yeast *iso*-1-cytochrome *c* and comparisons with other eukaryotic cytochromes *c*. *J Mol Biol* 214: 527-555.

S24. Tsukihara T, Shimokata K, Katayama Y, Shimada H, Muramoto K, et al. (2003) The low-spin heme of cytochrome *c* oxidase as the driving element of the proton-pumping process. *Proc Natl Acad Sci USA* 100: 15304-15309.

S25. Bushnell GW, Louie GV, Brayer GD (1990) High-resolution three-dimensional structure of horse heart cytochrome *c*. *J Mol Biol* 214: 585-595.

S26. Cunane LM, Barton JD, Chen ZW, Welsh FE, Chapman SK, et al. (2002) Crystallographic study of the recombinant flavin-binding domain of baker's yeast flavocytochrome b_2: comparison with the intact wild-type enzyme. *Biochemistry* 41: 4264-4272.
